# Aquaporin 1 promotes sensitivity of anthracycline chemotherapy in breast cancer by inhibiting β-catenin degradation to enhance TopoIIα activity

**DOI:** 10.1038/s41418-020-00607-9

**Published:** 2020-08-19

**Authors:** Wei Chong, Huikun Zhang, Zhifang Guo, Limin Yang, Ying Shao, Xiaoli Liu, Yawen Zhao, Zhe Wang, Ming Zhang, Caixia Guo, Li Fu, Yongjie Ma, Feng Gu

**Affiliations:** 1 Department of Breast Cancer Pathology and Research Laboratory, Tianjin Medical University Cancer Institute and Hospital, Tianjin, China; 2 Tianjin’s Clinical Research Center for Cancer, Tianjin Medical University Cancer Institute and Hospital, Tianjin, China; 3 Key Laboratory of Cancer Prevention and Therapy, Tianjin, China; 4 Key Laboratory of Breast Cancer Prevention and Therapy, Tianjin Medical University, Ministry of Education, Tianjin, China; 5 Department of Tumor Cell Biology, Tianjin Medical University Cancer Institute and Hospital, National Clinical Research Center for Cancer, Tianjin, China; 6 Department of Epidemiology and Biostatistics, Institute of Bioinformatics, University of Georgia, Athens, GA USA; 7 CAS Key Laboratory of Genomic and Precision Medicine, Beijing Institute of Genomics, Chinese Academy of Sciences, Beijing, China

**Keywords:** Predictive markers, Tumour biomarkers

## Abstract

Anthracyclines are a class of conventional and commonly used frontline chemotherapy drugs to treat breast cancer. However, the anthracycline-based regimens can only reduce breast cancer mortality by 20–30%. Furthermore, there is no appropriate biomarker for predicting responses to this kind of chemotherapy currently. Here we report our findings that may fill this gap by showing the AQP1 (Aquaporin1) protein as a potential response predictor in the anthracycline chemotherapy. We showed that breast cancer patients with a high level of AQP1 expression who underwent the anthracycline treatment had a better clinical outcome relative to those with a low level of AQP1 expression. In the exploration of the underlying mechanisms, we found that the AQP1 and glycogen synthase kinase-3β (GSK3β) competitively interacted with the 12 armadillo repeats of β-catenin, followed by the inhibition of the β-catenin degradation that led to β-catenin’s accumulation in the cytoplasm and nuclear translocation. The nuclear β-catenin interacted with TopoIIα and enhanced TopoIIα’s activity, which resulted in a high sensitivity of breast cancer cells to anthracyclines. We also found, the miR-320a-3p can attenuate the anthracycline’s chemosensitivity by inhibiting the AQP1 expression. Taken together, our findings suggest the efficacy of AQP1 as a response predictor in the anthracycline chemotherapy. The application of our study includes, but is not limited to, facilitating screening of the most appropriate breast cancer patients (who have a high AQP1 expression) for better anthracycline chemotherapy and improved prognosis purposes.

## Introduction

Breast cancer is the most common type of malignant tumor in women [1]. Anthracyclines are a class of conventional and widely applied frontline chemotherapy drugs to treat breast cancer. However, the anthracycline-based regimens can only reduce breast cancer mortality by 20–30% [2], and not all breast cancer patients benefit equally from this kind of chemotherapy. Currently, there is no appropriate biomarker for predicting responses to the anthracycline-based chemotherapy. Therefore, a reliable marker that could predict the sensitivity to anthracyclines and help screen the most appropriate patients to receive the anthracycline-based chemotherapies was desirable and highly valuable for individualized chemotherapy and improved patient survival.

To identify possible biomarkers in this aspect, we first randomly selected the invasive ductal carcinoma (IDC) tissues with their paired adjacent tissues from 22 patients’ samples. These patients included 10 epirubicin (EPI) sensitive and 12 EPI non-sensitive patients according to their primary tumor cells sensitivity to EPI. Through mRNA microarray analysis, we identified AQP1’s (Aquaporin1) involvement in the predictive response to anthracyclines’ sensitivity. Our clinical study of 341 IDC patients further confirmed that patients with a high AQP1 expression level had a better clinical outcome in the anthracycline chemotherapy relative to the patients with a low level of AQP1 expression. Previously, we reported that the membrane water channel protein AQP1 mainly localized in the cytoplasm of breast cancer cells and that the cytoplasmic expression of AQP1 could promote the malignant progression of breast cancer [3]. The expression pattern of AQP1 in breast cancer tissues, distinct from that in healthy tissues, suggests a possible relation between its cytoplasm localization and its function in breast cancer development.

In the present study, we discovered that the cytoplasmic AQP1 and GSK3β competitively interacted with the 12 armadillo repeats of β-catenin, a reaction that could inhibit the ubiquitin-proteasome degradation pathway of β-catenin in AQP1 overexpressed breast cancer cells. We also found that AQP1 could promote the accumulation of β-catenin in the cytoplasm so that the accumulated β-catenin can enter the nucleus. Once in the nucleus, β-catenin interacts with TopoIIα and enhances its catalytic activity, leading to a high sensitivity of breast cancer cells to anthracyclines [4–9]. Our study here suggests the usage of AQP1 as a response predictor in the anthracycline chemotherapy may be feasible. The application of our study includes, but is not limited to, facilitating screening of the most appropriate breast cancer patients (who have a high AQP1 expression) for better anthracycline chemotherapy and improved prognosis purposes.

## Materials and methods

### Ethics approval and consent to participate

Paraffin-embedded specimens from 341 patients diagnosed with IDC from 2004 to 2007 were randomly selected and reviewed from the archives of the Department of Breast Cancer Pathology and Research Laboratory, Tianjin Medical University Cancer Institute and Hospital. This study was approved by Institutional Ethics Committee of Tianjin Medical University Cancer Institute and Hospital (bc2017019), and each participant signed an informed consent document. Additionally, all animal work procedures were approved by the Ethics Committee of the Tianjin Medical University Cancer Institute and Hospital (bc2017019).

### Patients’ clinical information

Paraffin-embedded specimens from 341 patients diagnosed with IDC from 2004 to 2007 were randomly selected and reviewed from the archives of the Department of Breast Cancer Pathology and Research Laboratory, Tianjin Medical University Cancer Institute and Hospital. The histopathology and diagnosis in each case was confirmed independently by two pathologists according to the World Health Organization criteria for the classification of breast cancer. All 341 patients with IDC were women aged from 27 to 89 year (average age, 51.48 year). During the follow-up (range: 2–120 months), eight patients (2.3%) had recurrence, 53 patients (15.5%) had distant metastasis and 35 patients (10.3%) died of breast cancer. Among the 341 patients, the effect of AQP1 expression on prognosis was analyzed in the patients receiving CEF chemotherapy regimens (*n* = 97) and those who did not receive CEF chemotherapy regimens (*n* = 244), respectively. The effect of CEF regimens and CMF regimens (*n* = 59) on the prognosis of patients with high (*n* = 143) or low (*n* = 198) expression of AQP1 was also analyzed.

A total of 70 patients diagnosed with IDC from 2009 to 2013 in Tianjin Medical University Cancer Institute & Hospital were enrolled in this study. They were divided into EPI sensitive group and EPI non-sensitive group, according to collagen gel droplet-embedded culture drug sensitivity test (CD-DST). None of the patients had received neoadjuvant chemotherapy or preoperative radiation therapy before surgery.

### Collagen gel droplet-embedded culture drug sensitivity test procedure

The 70 patients who were diagnosed as IDC between July 2009 and February 2013 were randomly selected. CD-DST divided patients into two groups. EPI with low *T/C* ratio (*T/C* ratio ≤ 50%) represented the sensitive group and EPI with high *T/C* ratio (*T/C* ratio > 50%) represented the non-sensitive group. *T* was the total number of living cancer cells in the drug treatment group, *C* was the total number of living cancer cells in the control group. The *T* and *C* values were calculated as an average of triplicate droplets. Primary tumor cells were collected using Collagen Gel Culture kit Primaster (Nitta Gelatin Inc) [10, 11].

### Microarray hybridization and computational analysis

Breast cancer tissues and their paired adjacent tissues were obtained from 10 EPI sensitive and 12 EPI non-sensitive breast cancer patients according to primary tumor cells sensitivity to EPI. The human materials were obtained with informed consent, and the study was approved by the Clinical Research Ethics Committee. None of patients received neoadjuvant chemotherapy before this study. The global LncRNA and mRNA expression profiling and data analysis for these tissues were obtained through microarray analysis by Agilent human LncRNA microarray 4 × 180 K gene expression data (Bioassay Laboratory of CapitalBio Corporation, Beijing, China). Genes with *P* < 0.05 and |fold change| > 2 were considered as differentially expressed genes (DEGs). DEGs in the EPI sensitive group and EPI non-sensitive group by mRNA microarray analysis were listed in the Supplementary Table S1.

### Bioinformatics analysis

The 1218 breast cancer patients’ RNAseq data showed in Fig. 3a, b and Supplementary Fig. S3a, b was obtained from the TCGA (https://portal.gdc.cancer.gov), and the gene expression profiles were divided into two groups according to the expression of AQP1. The limma R package was used to calculate the differentially expressed statistics. We used these statistics as input to R-function in ClusterProfile package to perform GSEA [12].

The miRNA expression profile and doxorubicin sensitivity (IC_50_) data of breast cancer cell lines (*n* = 44), shown in Supplementary Fig. S7a, were downloaded from Cancer Cell Lines Encyclopedia (CCLE, https://portals.broadinstitute.org/ccle). We divided breast cancer cell lines (*n* = 44) into two groups (miR-320a-3p high group and miR-320a-3p low group) by using the median expression level as cutoff point. The relationship of miR-320a-3p level and doxorubicin sensitivity (IC_50_) was analyzed by correlations analysis.

The miRNA and mRNA gene expression profile of breast cancer tissues (*n* = 98) summarized in Supplementary Fig. S7b–c were downloaded from GEO (ID: GSE19783). The relationship of miR-320a-3p and AQP1 expression were analyzed by correlations analyses.

### Cell culture and reagents

MDA-MB-231 cells and HEK-293T cells were cultured in DMEM medium supplemented with 10% fetal bovine serum in a 5% CO_2_ incubator at 37 °C. T47D cells, MCF7 cells were obtained from the ATCC and cultured in RPMI-1640 medium supplemented with 15% fetal bovine serum in a 5% CO_2_ incubator at 37 °C. Cells were tested and authenticated in Beijing Microread Genetics Co., Ltd. (Beijing, China) by short tandem repeat (STR) profiling. All cell lines tested negative for mycoplasma contamination. MG132 (C2211) and CHX (C1998) were purchased from Sigma. Recombinant human Wnt-3a was purchased from R&D Systems. The transfection of miR-320a-3p mimic, miR-144-3p mimic, and miR-29a-3p mimic with miRNA control (miR-NC) (GenePharma, China) were performed according to the manufacturer’s instruction using Lipofectamine 3000 reagent (Invitrogen). The final concentration of miRNA was 20 nM. miRNA sequences are listed in Supplementary Table S2.

### Plasmid construction and transfection

Plasmids and RNA interference sequences are listed in Supplementary Table S2. Full length of molecules or its mutants were amplified by PCR using primers (GenBank). Molecules or mutants with a GFP label, HA label, or 3×Flag label were cloned into pCDH-CMV-MCS-EF1-Puro lentiviral vector (http://www.addgene.org/) or pcDNA TM 3.1(+) (InvitrogenTM, V790-20) [13]. The sequences of the inserts were 100% correct. TopoIIα and β-catenin-specific shRNAs and scrambled sequence were synthesized and cloned into pLKO.1 pure vector, respectively. Lentiviruses were produced by co-transfection of a lentiviral plasmid, packing plasmids ΔR and pVSVg into HEK-293T cells. After transfection, supernatant was collected and the virus was used to infect cells. Stable lentivirus-infected cells were selected with puromycin or G418 and verified by western blot analysis.

The control siRNA and siβ-catenin were synthesized at GenePharma Inc. (Shanghai, China). The siRNA oligonucleotides were transfected into cells using X-tremeGENE HP DNA Transfection Reagent (Roche) following the manufacturer’s recommendations. The interference efficiency of siRNA was routinely assessed by western blot at 48 h after transfection.

### Immunohistochemistry analysis and evaluation

Sections (5 μm thick) were dewaxed, hydrated, and heated for antigen retrieval. They were blocked with hydrogen peroxide and normal goat serum, and subsequently incubated overnight with AQP1, β-catenin, or TopoIIα. Antibodies used in this study are listed in Supplementary Table S3. All sections were stained with 3,3′-diaminobenzidinetetra-hydrochloride (DAB) [14].

AQP1, β-catenin and TopoIIα were evaluated individually according to the H score system, which were based on the staining intensity and the percentage of cells stained positively. Stained tissue sections were blind reviewed by two pathologists based on a double scoring system (staining intensity multiplied by staining area) [15]. Staining intensity for AQP1, β-catenin, and TopoIIα were scored as follows: 0-no staining, 1-definite but weak staining, 2-moderate staining, and 3-strong staining. The positive staining area of AQP1 and β-catenin was scored as follows: 0 (0%), 1 (1–49%), 2 (50–75%), and 3 (76–100%), and produced a total score range of 0–9. Because the nuclear TopoIIα staining was present in a uniform intensity but to different extent, this nuclear expression was assessed by the percentage of positively nucleic-stained cells and scored on a scale of 0 or 1. In our present study, the low and high expression of AQP1 and β-catenin were samples with score 0–2 and score 3–9, respectively. Low and high expression of TopoIIα was regarded as score 0 and score 1, respectively.

### Antibodies and western blot

The cells or breast cancer tissues were lysed in ice-cold lysis buffer, then the lysed proteins were separated by SDS-PAGE gel, followed by transferring onto a BioTrace NT Nitrocellulose Transfer Membrane (Pall Corp. Biolab 66485). Antibodies used in this study are listed in Supplementary Table S3. Afterwards, secondary antibodies (IRDye®800CW) were used to incubate with the membrane. Blots were visualized using the Odyssey imaging system (Li-Cor Biosciences, Lincoln, NE, USA).

### Immunofluorescence analysis

Pretreated sterile coverslips were placed in the 12-well plate, then cells were plated in each well followed by 24-h incubation in the incubator. Afterwards, the cells were fixed and permeabilized before being incubating with primary antibodies overnight at 4 °C. Antibodies used in this study are listed in Supplementary Table S3. After being washed three times with PBS, the cells were stained with the secondary antibody (Invitrogen, New York, USA) at the room temperature for 1 h in a dark box. DAPI (Solarbio, Beijing, China) was used to stain the nuclei. The fluorescence of cells was visualized with a confocal laser scanning microscope (Leica TCS SP5) or fluorescence microscope (Carl Zeiss). The fluorescence intensity was measured in ImageJ software [16]. All the immunofluorescence and microscopy experiments were performed blinded.

### Co-immunoprecipitation assay

The co-immunoprecipitation (Co-IP) was performed essentially the same as previously described [15, 17]. Briefly, breast cancer tissues or cells lysates were obtained in Co-IP lysis buffer (pH 7.4) and gently rotated at 4 °C overnight followed by centrifuging at 12,000 rpm for 10 min. Then the supernatant was immunopurified with anti-flag M2 affinity gel (A2220, Sigma) and eluted with flag peptides. The eluates were resuspended in sample buffer and boiled for 5 min for western blot examination.

Cell lysates were gently rotated at 4 °C overnight followed by centrifuging. Nonspecific protein was removed by adding Protein A (sc-2001, Santa cruz), the mixture was centrifuged and the supernatant was divided into two groups by using antibodies or control IgG. Finally, the precipitates were subjected to western blot to examine the expression of target protein.

### MTT assay and cell ATP/viability assay

Cells were seeded and incubated in 24-well plates with three replicates. At the final incubation period, viable cells were quantified using MTT. The MTT stock solution (5 mg/ml) was added to each well. After incubated for 4 h in the incubator, medium was removed and the converted dye was solubilized with DMSO. The absorbance of the converted dye was measured at a wavelength of 570 nm [18]. And viable cells were also used the CellTiter-Glo luminescent cell viability assay kit (Promega, Madison, WI, USA) to measure the ATP levels as manufacturer’s description.

### miRNA target prediction

TargetScan (http://www.targetscan.org/), miPDB (http://mirdb.org/cgi-bin/search.cgi), and miRanda (http://www.microrna.org/microrna/home.do) were used to predict the candidate miRNAs may interact with 3′-UTR of AQP1.

### RNA extraction and quantitative real-time qPCR

RNA preparation and RT-qPCR assays were assembled as described using gene-specific primers listed in Supplementary Table S4. The total RNA was isolated from cells using Trizol reagent (Life technologies, Carlsbad, CA, USA) according to the manufacturer’s instructions. cDNA was generated by the RTase M-MLV (TaKaRa, China) as described in the manufacturer’s protocol. Quantitation of all gene transcripts was done by qPCR using SYBR Green PCR Master Mix (TaKaRa, China), and the expression of GAPDH was used as the internal control. Fold changes were calculated using the ΔΔCt method in Microsoft Excel [16].

### Ubiquitylation assay

The cells were treated with 1 μM of MG132 (Sigma C2211) for 6 h prior to harvesting. Then cells were washed three times with ice-cold PBS and cell resuspended with Co-IP lysis buffer (pH 7.4) plus protease inhibitors (Roche). Cell lysates were gently rotated at 4 °C overnight followed by centrifuging. Nonspecific protein was removed by adding Protein A (sc-2001, Santa cruz), the mixture was centrifuged and the supernatant was divided into two groups by using β-catenin antibodies (Abcam32572) or control IgG. Finally, the precipitates were subjected to western blot to examine the expression of ubiquitin antibody [17].

### Preparation of cytosol/nuclear extract and subcellular fractionation

Cytoplasmic and nuclear extracts were prepared by the Nuc-Cyto-Mem Preparation Kit (P1201, Applygen Technologies, Beijing, China). In brief, cells were lysed by Dounce homogenization with CER buffer on ice. Then, the whole-cell lysate was centrifuged at 800 × *g* for 5 min at 4 °C. The pellet (nuclear component) was washed with the ice-cold NER buffer, clarified by low-speed centrifugation and collected as nuclei. The supernatant of whole-cell lysate was centrifuged at 4000 × *g* for 5 min at 4 °C three times and the supernatant was collected as cytoplasmic fraction. The isolated protein fractions were analyzed by western blot [15].

### TOP/FOP flash

Cells were plated on the white-bottomed 24-well plates with three replicates. Cells were serum-starved overnight and co-transfected with 0.2 μg TOP flash or FOP flash expression plasmids were a gift from Prof. Xiaoguang Liu (Department of Orthopaedics, Peking University Third Hospital, China) and 0.1 μg pRL-TK (*Renilla* TK-luciferase vector; Promega Corp.) as an internal control, using X-tremeGENE HP DNA Transfection Reagent (Roche). After 48 h, the luciferase activity was measured with the Dual-Luciferases Reporter Assay kit (Promega E1980) according to manufacturer’s protocols. Firefly luciferase activity was normalized for transfection efficiency by dividing the results by the *Renilla* luciferase activity. The TOP/FOP relative fold was used as a measure of β-catenin-driven transcription.

### TopoGen decatenation assay

TopoIIα enzymatic activity was assayed by measuring the decatenation of kinetoplast (k)-DNA (TopoGen). A standard assay carried out in a total volume of 20 μl included 50 mM Tris HCl, pH 7.9, 88 mM KCl, 10 mM MgCl_2_, 0.5 mM EDTA, 10 mM ATP, 10 mM DTT, 100 μg/ml BSA, and 300 ng of kDNA. The reaction mixture containing TopoIIα extracted from cells was incubated at 37 °C, and the reaction was stopped by the addition of 5 μl of stop solution (5% SDS, 25% Ficoll, and 0.05% bromophenol blue). The samples were resolved by electrophoresis at 130 V using a 1% agarose gel in Tris-acetate-EDTA buffer with 0.5 μg/ml ethidium bromide (EB) and photographed under UV illumination [19, 20].

### Animal tumor transplantation and therapy

The BALB/c nude mice were purchased from Nanjing model animal research center. All animal work procedures were approved by the Ethics Committee of the Tianjin Medical University Cancer Institute and Hospital. MDA-MB-231 or AQP1/MDA-MB-231 cells were subcutaneously inoculated into the third pair of fat pads on the right side of the nude mice (female, 4 weeks old) at a density of 5 × 10^6^ in 0.2 ml of PBS. Tumors were measured by using a caliper and were weighted. The tumor volume was calculated by: (large diameter) × (small diameter)^2^/2. Then orthotopically implanted tumors were formed. When the volume of the tumor was enough to be inoculated, MDA-MB-231 or AQP1/MDA-MB-231 tumors were randomly chosen and cut into small pieces (2 × 2 mm) and subsequently anchored to the third pair of fat pads on the right side of new nude mice (*n* = 40/group). When the average volume of tumors reached 180 mm^3^, the tumor-bearing mice were randomized into treatment groups and received 8 mg/kg of EPI or 10 mg/kg of MTX (Methotrexate) via the intraperitoneal route each week, while the control group was injected with the same volume of 0.9% NS (normal saline). The tumor growth and tumor-bearing survival rates were monitored daily. Tumors were carefully removed from the mice, washed, and placed in cold PBS. Then the tumor was fixed with 4% paraformaldehyde. Samples were soaked in wax, and then cut to 5-μm-thick sections with routine histological methods [21].

### Statistical analyses

The SPSS Version 20.0 software package was used for statistical analysis. Mann–Whitney *U* test, ANOVA test, and *χ*^2^ test were performed for group comparisons. Correlations between two variables were evaluated by Spearman’s Rank-Correlation test or Pearson correlation analysis. Overall survival (OS) and progression-free survival (PFS) rates were assessed using the Kaplan–Meier method, and the log-rank test was applied to compute *P* values. For analysis of in vitro cellular experiments, statistical significance for comparisons between groups was determined using a two-tailed Student’s *t*-test. All data was presented as mean ± SEM. Three independent experiments were performed. A two-sided *P* < 0.05 was considered statistically significant in all analysis.

## Results

### mRNA microarray combined with bioinformatics analysis and clinical analysis demonstrated that AQP1 upregulated EPI chemosensitivity

In order to identify DEGs and specific pathways involved in EPI sensitivity, we applied a mRNA microarray to generate specific sets of genes at genome-wide in tumor tissues with their paired adjacent tissues from 22 IDC patients’ samples. These patients included 10 EPI sensitive and 12 EPI non-sensitive breast cancer patients according to their primary tumor cells sensitivity to EPI.

**Fig. 1:**
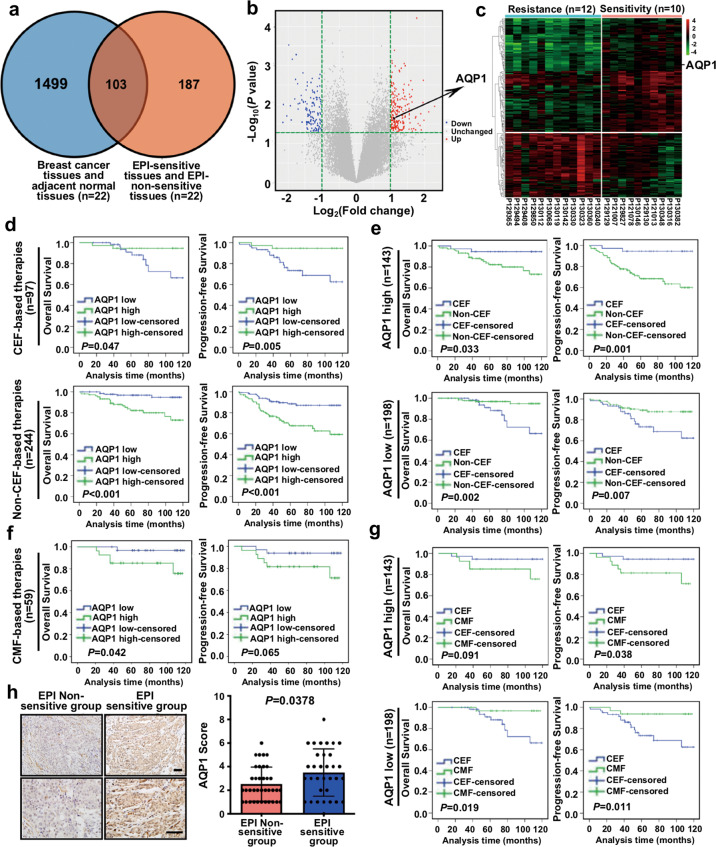
mRNA microarray combined with bioinformatics analysis and clinical analysis demonstrated that AQP1 upregulated EPI chemosensitivity. **a** The Venn diagram showed DEGs in breast cancer tissues and their paired adjacent tissues. DEGs in EPI sensitive group and non-sensitive group, along with 103 overlapped DEGs were also shown. Genes with *P* < 0.05 and |fold change| > 2 were considered as DEGs. **b** The volcano plot: green, red, and black points represented downregulated (*n* = 105), upregulated (*n* = 185), and no statistically significant difference genes, respectively, in the EPI non-sensitive group versus the EPI sensitive group. *X*-axes showed log_2_ (fold change) and *y*-axes showed −log_10_ (*P* value). **c** The heatmap and cluster analysis: EPI non-sensitive group: blue, EPI sensitive group: pink. Red denoted high expression and green represented low expression. The high expression of AQP1 in EPI sensitive group was shown. Not all DEGs in the figure were labeled. **d** OS (left panel) and PFS (right panel) curves of IDC patients treated with CEF-based therapies (upper panel) or non-CEF-based therapies (lower panel) with AQP1 expression (log-rank test). **e** OS (left panel) and PFS (right panel) curves of patients with high expression of AQP1 (upper panel) or low expression of AQP1 (lower panel) who received CEF-based therapies or non-CEF-based therapies (log-rank test). **f** OS (left panel) and PFS (right panel) curves of IDC patients treated with CMF-based therapies with AQP1 expression (log-rank test). **g** OS (left panel) and PFS (right panel) curves of patients with high expression of AQP1 (upper panel) or low expression of AQP1 (lower panel) who received CEF-based therapies or CMF-based therapies (log-rank test). **h** Representative immunohistochemical images of AQP1 expression in the EPI non-sensitive group (*n* = 37) and the EPI sensitive group (*n* = 33), respectively. Scale bars: 100 μm, (left panel). The AQP1 expression was higher in the EPI sensitive group than the EPI non-sensitive group (Mann–Whitney *U* test, *P* = 0.0378; right panel).

First, we analyzed DEGs in IDC tissues and adjacent tissues, and DEGs in EPI sensitive group and EPI non-sensitive group, respectively. The Venn diagram showed 103 overlapped DEGs that included AQP1, CTLA4, ADH1A, etc (Fig. 1a). The volcano plot, Heatmap, and cluster analysis illustrated that the expression of AQP1 was higher in the EPI sensitive group relative to the EPI non-sensitive group (Fig. 1b, c). Then these DEGs were analyzed in Gene Set Enrichment Analysis (GSEA), Gene Oncology (GO), and Kyoto Encyclopedia of Genes and Genomes (KEGG) databases. We found, a plenty of pathways and functions were significantly enriched, such as the channel activity, cell proliferation, cell migration, PI3K-Akt signaling pathway, and MAPK signaling pathway, most of these functions and pathways were mediated by AQP1 (Supplementary Fig. S1a–e). Therefore, these results suggested an important role of AQP1 in the upregulation of EPI chemosensitivity.

Next, we analyzed 341 IDC patients with a complete clinical follow-up. We did not find any difference in clinicopathologic characteristics between CEF and non-CEF-based chemotherapy patients at the baseline (Supplementary Table S5). Then we analyzed the relation between the AQP1 expression and prognosis of patients treated with conventional CEF (cyclophosphamide, anthracyclines, and 5-fluorouracil)-based chemotherapy. The high expression of AQP1 indicated a longer period of OS and PFS (OS: *P* = 0.047, PFS: *P* = 0.005) in patients treated with CEF-based chemotherapy. However, patients with AQP1 high expression in non-CEF-based chemotherapy showed a significantly shorter period of OS and PFS (OS: *P* < 0.001, PFS: *P* < 0.001; Fig. 1d). In addition, when the 341 IDC cases were divided into the AQP1 low-expression group and the AQP1 high-expression group, we also did not find any difference between two groups in clinicopathologic characteristics at the baseline (Supplementary Tables S6 and S7). In the AQP1 high expression group, both OS and PFS (OS: *P* = 0.033, PFS: *P* = 0.001) of breast cancer patients treated with CEF regimens were significantly longer than those with non-CEF regimens, and patients with low AQP1 expression could not benefit from CEF regimens (Fig. 1e). We further analyzed the relation between the AQP1 expression and prognosis of patients treated with CMF (cyclophosphamide, methotrexate, and 5-fluorouracil) regimens. We found that breast cancer patients with a high AQP1 expression could not benefit from CMF regimens (Fig. 1f). Moreover, in the AQP1 high expression group, PFS (*P* = 0.038) of breast cancer patients treated with CEF regimens was significantly longer than those treated with CMF regimens, and patients with the low AQP1 expression could not benefit from CEF regimens (Fig. 1g).

In CEF-based therapies, the percentage of high AQP1 expression in patients who developed recurrence or metastasis within 5 years was significantly lower than those who were progression-free within 5 years (*P* = 0.032, Supplementary Fig. S2a). However, in CMF-based therapies, there was no significant difference (Supplementary Fig. S2b). Furthermore, we found that in CEF-based therapies, the expression of AQP1 in patients who presented disease progression (recurrence, metastasis, or death) within 5 years was lower than patients who were progression-free within 5 years (*P* = 0.0274, Supplementary Fig. S2c). Likewise, no statistical difference was observed in CMF-based therapies (Supplementary Fig. S2d). We also applied immunohistochemical staining in another cohort of IDC patients (*n* = 70), which were divided into the EPI sensitive group (*n* = 33) and the EPI non-sensitive group (*n* = 37) by primary tumor cells’ sensitivity to EPI. The expression of AQP1 in the EPI sensitive group was found higher than that in the EPI non-sensitive group (*P* = 0.0378; Fig. 1h and Table 1). Altogether, these results demonstrated that AQP1 upregulated EPI chemosensitivity.

**Table 1:** AQP1 expression in the EPI non-sensitive group and the EPI sensitive group.

	AQP1 expression, *n* (%)		*χ*^2^	*P* value
	Low (0–2)	High (3–9)		
EPI sensitive group	10 (30.3)	23 (69.7)	5.975	0.015
EPI non-sensitive group	22 (59.5)	15 (40.5)	–	–

*P* value was calculated by *χ*^2^ test.

### High AQP1 expression elevated EPI chemosensitivity in vitro and in vivo

**Fig. 2:**
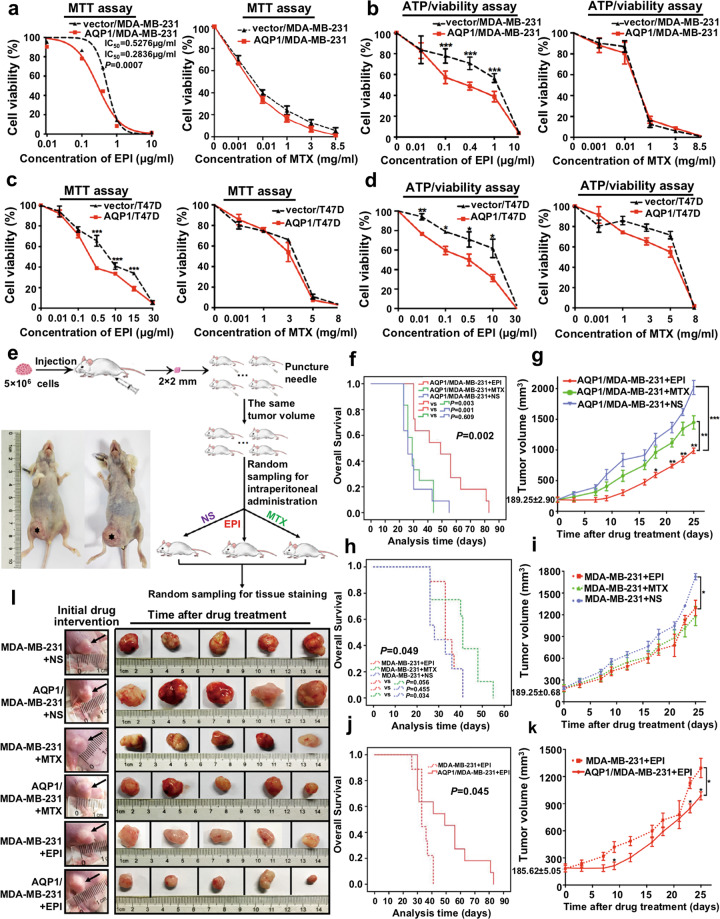
High AQP1 expression elevated EPI chemosensitivity in vitro and in vivo. **a**, **b** Cell viability of vector/MDA-MB-231 and AQP1/MDA-MB-231 with different concentration of EPI (left panel) or MTX (right panel) treatment for 48 h was tested by MTT assay (**a**) and ATP/viability assay (**b**). Values were expressed as mean ±  SEM from three independent experiments (two-tailed Student’s *t* test, ***P < 0.001). **c**, **d** Cell viability of vector/T47D and AQP1/T47D with different concentration of EPI (left panel) or MTX (right panel) treatment for 48 h was tested by MTT assay (**c**) and ATP/viability assay (**d**). Values were expressed as mean ±  SEM from three independent experiments (two-tailed Student’s *t* test, *P < 0.01, **P < 0.01, ***P < 0.001). **e** Description of timeline and treatment of the tumor xenografts mouse model. Representative pictures of mice xenografts were captured after 3 weeks of tumor-bearing (left panel). **f** The survival was evaluated in AQP1/MDA-MB-231 mice groups treated with NS (blue), MTX (green) or EPI (red) (log-rank test). **g** Volume of tumor xenografts in AQP1/MDA-MB-231 mice groups treated with NS (blue), MTX (green) or EPI (red) (two-tailed Student’s *t* test and two-way ANOVA, *P < 0.05, **P < 0.01, ***P < 0.001). **h** The survival was evaluated in MDA-MB-231 mice groups treated with NS (blue), MTX (green), or EPI (red) (log-rank test). **i** Volume of tumor xenografts in MDA-MB-231 mice groups treated with NS (blue), MTX (green), or EPI (red) (two-tailed Student’s *t* test and two-way ANOVA, *P < 0.05). **j** The survival was evaluated in MDA-MB-231 and AQP1/MDA-MB-231 mice groups treated with EPI (log-rank test). **k** Volume of tumor xenografts in MDA-MB-231 and AQP1/MDA-MB-231 mice groups treated with EPI (two-tailed Student’s *t* test and two-way ANOVA, *P < 0.05). **l** The representative images of tumor size of each group were captured. Arrowheads denoted the tumor status of mice with initial drug intervention.

First, the role of AQP1 in the regulation of EPI sensitivity was validated in vitro. The endogenous AQP1 expression was detectable in mouse kidney tissues and breast cancer tissues, but it was undetectable in parental MDA-MB-231, T47D, and MCF7 cell lines. Then, we overexpressed AQP1 in MDA-MB-231 and T47D cells and detected its exogenous expression in western blot (Supplementary Fig. S2e–f). Next, we detected the sensitivity of vector/MDA-MB-231 and AQP1/MDA-MB-231 cells to EPI and MTX by MTT assay and ATP/viability assay, respectively. It is worth noting that AQP1/MDA-MB-231 cells were more sensitive to EPI than the vector/MDA-MB-231 cells, but there was no significant difference in these cells with MTX treatment (Fig. 2a, b). Similar results were also found in vector/T47D and AQP1/T47D cells (Fig. 2c, d).

Next, we validated the in vitro results in in vivo experiments. Figure 2e showed the timeline and treatment of the tumor xenografts mouse model. The MDA-MB-231 and AQP1/MDA-MB-231 mice groups were treated with EPI, MTX, or NS, respectively. We observed the tumor formation and survival of the mice. The EPI-treated group showed the longest survival time and the minimum xenografts in AQP1/MDA-MB-231 mice (Fig. 2f, g), while no difference was found among the three treatments in the MDA-MB-231 mice (Fig. 2h, i). The AQP1/MDA-MB-231+EPI mice group had a better prognosis and smaller xenografts than the MDA-MB-231+EPI mice group, which further confirmed that high AQP1 expression was more sensitive to EPI (Fig. 2j, k). The representative xenografts of each group were shown in Fig. 2l. These findings together provided strong in vitro and in vivo evidence that high AQP1 expression elevates the EPI chemosensitivity.

### AQP1 interacted with β-catenin and patients with both high expression of AQP1 and β-catenin presented the best outcomes with the anthracycline chemotherapy

**Fig. 3:**
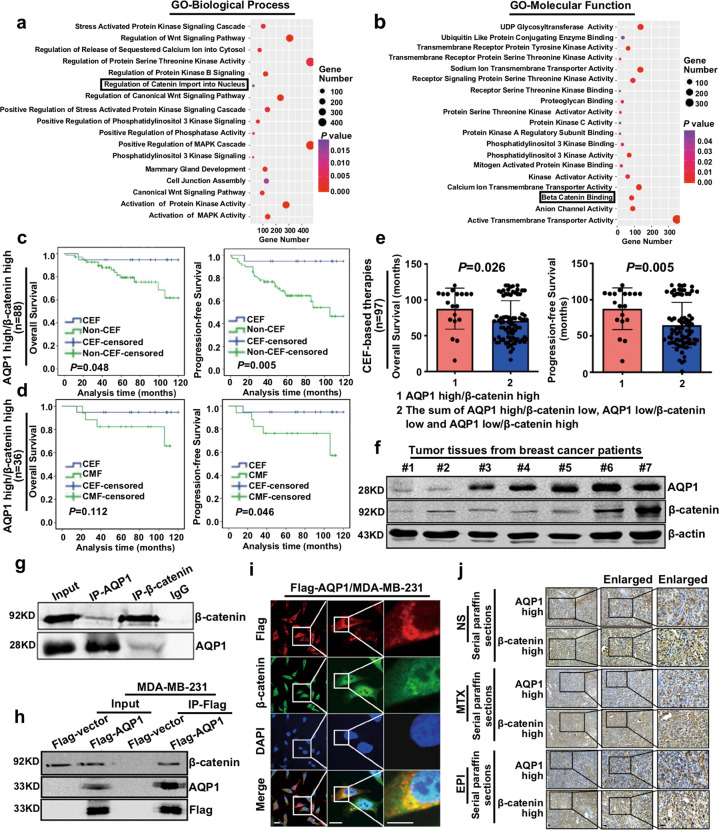
AQP1 interacted with β-catenin and patients with both high expression of AQP1 and β-catenin presented the best outcomes with the anthracycline chemotherapy. **a**, **b** The 1218 breast cancer patients’ RNAseq data was divided into two groups according to the expression of AQP1. The limma R package was used to calculate the differentially expressed statistics. GO functional annotation genes in the module obtained by GSEA. *Y*-axis showed the GO terms and *x*-axis showed the gene number of each term, the degree of color represented *P* value. **c** Patients were divided into four subgroups according to the expression of both AQP1 and β-catenin. The AQP1 high/β-catenin high subgroup patients who received CEF-based therapies had a longer OS (left panel) and PFS (right panel) than non-CEF regimens (log-rank test). **d** The AQP1 high/β-catenin high group who received CEF-based therapies had a longer PFS (right panel) than CMF regimen patients (log-rank test). **e** The AQP1 high/β-catenin high group showed a longer OS (left panel) and PFS (right panel) in CEF-based therapies patients (*n* = 97) than the rest (i.e., the sum of AQP1 high/β-catenin low, AQP1 low/β-catenin low, and AQP1 low/β-catenin high; two-tailed Student’s *t* test). **f** The expression of AQP1 and β-catenin was detected by western blot using tumor tissues from breast cancer patients. β-actin was the loading control. **g**, **h** Co-immunoprecipitation results of AQP1 and β-catenin in breast cancer tissues (**g**) and Flag-AQP1/MDA-MB-231 cells (**h**). **i** Co-localization of Flag-AQP1 and β-catenin in Flag-AQP1/MDA-MB-231 cells. Insets showed a high-magnification view of the indicated region. Scale bars: 100 μm. **j** The expression of AQP1 and β-catenin was detected by immunohistochemistry analysis of serial paraffin sections in mice tumor tissues. Scale bars: 200 μm. Experiments (**f**–**i**) were independently repeated for three times.

We selected 1218 breast cancer patients’ RNAseq data from TCGA to further explore how AQP1 modulated EPI sensitivity. Then, the gene expression profile was divided into two groups according to the level of AQP1. GSEA analysis showed AQP1 was involved in β-catenin binding and regulation of β-catenin import into nucleus (Fig. 3a, b and Supplementary Fig. S3a, b). Next, we detected the expression of β-catenin in 341 IDC patients by immunohistochemistry. The clinical analysis results showed that β-catenin may not be involved in the regulation of anthracyclines sensitivity (Supplementary Fig. S3c–f). Then, we divided the 341 IDC patients into four subgroups (AQP1 high/β-catenin high, AQP1 high/β-catenin low, AQP1 low/β-catenin high, and AQP1 low/β-catenin low) utilizing combination analysis of AQP1 and β-catenin expression. In the AQP1 high/β-catenin high subgroup, patients who received CEF-based therapies had a better prognosis than patients treated with non-CEF-based therapies (OS: *P* = 0.048, PFS: *P* = 0.005; Fig. 3c). Similarly, the AQP1 high/β-catenin high subgroup patients who received CEF-based therapies had a longer PFS than CMF-based therapies (*P* = 0.046; Fig. 3d). In addition, the AQP1 low/β-catenin low subgroup patients who received CEF-based therapies had a shorter OS and PFS than those treated with non-CEF regimens (OS: *P* = 0.008, PFS: *P* = 0.034) or CMF regimens (OS: *P* = 0.038, PFS: *P* = 0.040). However, there was no significant difference in OS or PFS between AQP1 low/β-catenin high subgroup and AQP1 high/β-catenin low subgroup patients (Supplementary Fig. S4a, b). In CEF-based therapies, AQP1 high/β-catenin high subgroup patients had the best survival outcome (OS: *P* = 0.026, PFS: *P* = 0.005; Fig. 3e).

We further validated the relation between AQP1 and β-catenin. The AQP1 expression was found positively correlated with β-catenin in 341 IDC patients (Table 2). The western blot and serial paraffin sections analysis of IDC tissues rendered similar results (Fig. 3f and Supplementary Fig. S4c). Our results also showed AQP1 and β-catenin co-immunoprecipitated with each other in breast cancer tissues (Fig. 3g) and Flag-AQP1/MDA-MB-231 cells (Fig. 3h), respectively. In addition, AQP1 and β-catenin co-localized in the cytoplasm of Flag-AQP1/MDA-MB-231 cells and mGFP-AQP1/MDA-MB-231 cells (Fig. 3i and Supplementary Fig. S4d, e). AQP1 expression was found positively correlated with β-catenin in serial paraffin-embedded sections of mice tumor tissues (Fig. 3j). As a consequence, AQP1 interacted with β-catenin in breast cancer cells, and only patients with both high expression of AQP1 and β-catenin presented the best outcomes in the anthracycline chemotherapy.

**Table 2:** The association of AQP1 and β-catenin in IDC patients.

	*n*	AQP1 expression, *n* (%)		*r*_s_	*P* value
		Low expression (0–2)	High expression (3–9)		
β-catenin expression					
Low expression (0–2)	156	101 (64.7)	55 (35.3)	0.124	0.022
High expression (3–9)	185	97 (52.4)	88 (47.6)	–	–

*P* value was calculated by Spearman’s Rank-Correlation test.

### The C-terminal tail of AQP1 and GSK3β competitively interacted with the 12 armadillo repeats of β-catenin, then inhibited β-catenin degradation

**Fig. 4:**
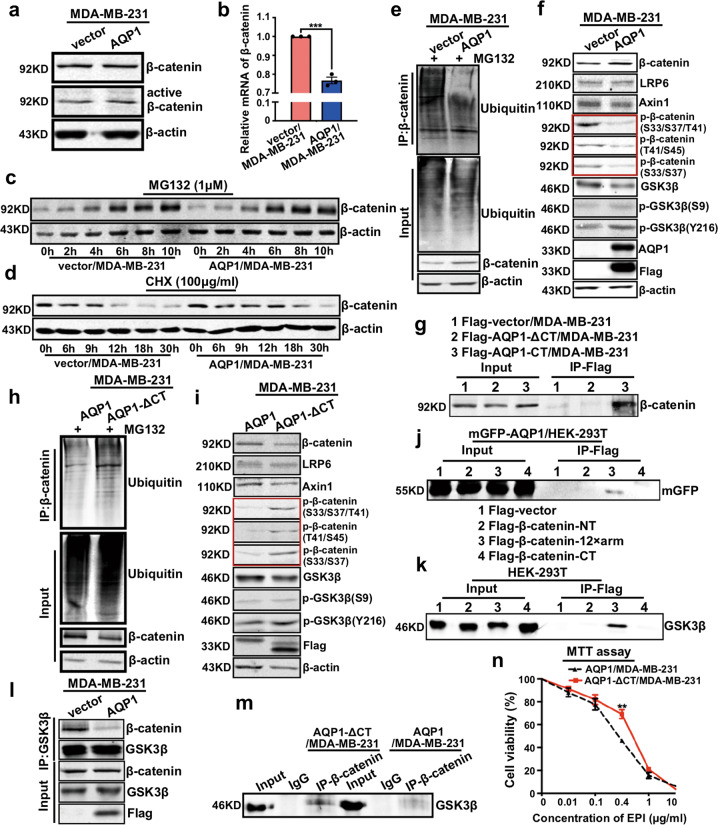
The C-terminal tail of AQP1 and GSK3β competitively interacted with the 12 armadillo repeats of β-catenin, then inhibited β-catenin degradation. **a** Western blot analyses of β-catenin and active β-catenin in vector/MDA-MB-231 and AQP1/MDA-MB-231 cells, β-actin was the loading control. **b** RT-qPCR results of mRNA level of β-catenin in vector/MDA-MB-231 and AQP1/MDA-MB-231 cells. GAPDH was used as control. Values were expressed as mean ± SEM (two-tailed Student’s *t* test, ***P < 0.001). **c**, **d** Vector/MDA-MB-231 and AQP1/MDA-MB-231 cells were treated with 1 μM MG132 (**c**) or 100 μg/ml CHX (**d**) and harvested at the noted time points followed by western blot analyses. Here the β-actin was the loading control. **e** Vector/MDA-MB-231 and AQP1/MDA-MB-231 cells were extracted and immunoprecipitated with β-catenin antibody and then immunoblotted with ubiquitin antibody, β-actin was the loading control. **f** Western blot of lysates of vector/MDA-MB-231 and AQP1/MDA-MB-231 cells were analyzed with indicated antibodies, β-actin was the loading control. **g** Co-immunoprecipitation with Flag antibody showed the interaction of Flag-AQP1-ΔCT or Flag-AQP1-CT with β-catenin. **h** AQP1/MDA-MB-231 and AQP1-ΔCT/MDA-MB-231 cells were extracted and immunoprecipitated with β-catenin antibody and then immunoblotted with ubiquitin antibody, β-actin was the loading control. **i** Western blot of lysates of AQP1/MDA-MB-231 and AQP1-ΔCT/MDA-MB-231 cells were analyzed with indicated antibodies, β-actin was the loading control. **j** 12 armadillo repeats (12 × arm) of β-catenin mediated the interaction of β-catenin with AQP1 in co-immunoprecipitation experiments. **k** 12 armadillo repeats (12 × arm) of β-catenin mediated the interaction of β-catenin with GSK3β in co-immunoprecipitation experiments. **l** Endogenous GSK3β was immunoprecipitated. The interaction of GSK3β with β-catenin was analyzed by immunoblotting for β-catenin in MDA-MB-231 cells in the presence or absence of AQP1. Whole-cell lysates were directly subjected to western blot using Flag, GSK3β, and β-catenin antibodies as input. **m** Endogenous β-catenin was immunoprecipitated and interaction of GSK3β with β-catenin was analyzed by immunoblotting for GSK3β in Flag-AQP1/MDA-MB-231 and Flag-AQP1-ΔCT/MDA-MB-231 cells. **n** Cell viability of AQP1/MDA-MB-231 and AQP1-ΔCT/MDA-MB-231 with different concentration of EPI treatment for 48 h was tested in MTT assay. Values were expressed as mean ± SEM (two-tailed Student’s *t* test, **P < 0.01). All Experiments were independently repeated for three times.

Active β-catenin is an active form of β-catenin that is dephosphorylated on Ser37 or Thr41 and is not degraded by degradation complexes [22]. We examined the expression of β-catenin and active β-catenin in western blot and found no significant difference between the vector/MDA-MB-231 and AQP1/MDA-MB-231 cells (Fig. 4a and Supplementary Fig. S5a). However, the mRNA level of β-catenin was downregulated in AQP1/MDA-MB-231 cells (Fig. 4b). Subsequently, we used the proteasome inhibitor MG132 to examine whether AQP1 regulates the abundance of β-catenin. We found the expression of β-catenin gradually accumulated with a longer MG132 treatment time, but no significant difference was noticed between the vector/MDA-MB-231 and AQP1/MDA-MB-231 cells (Fig. 4c, Supplementary Fig. S5b). Next, we applied CHX (inhibitor of protein synthesis) to detect whether AQP1 affects β-catenin degradation. As Fig. 4d showed, AQP1 inhibited β-catenin degradation and the expression of β-catenin decreased to 50% after CHX treatment needed a longer time in the AQP1 overexpression group than the control (*t*_1/2_ = 10.98 h vs *t*_1/2_ = 20.79 h; Supplementary Fig. S5c).

We next investigated whether the AQP1-promoted β-catenin stabilization is a consequence of AQP1-catalyzed β-catenin deubiquitination. We discovered that the level of ubiquitination of β-catenin was reduced in AQP1/MDA-MB-231 cells (Fig. 4e). Actually, the cytoplasmic β-catenin can be efficiently captured by the destruction complex. As a member of the destruction complex, GSK3β can phosphorylate β-catenin at Ser33, Ser37, and Thr41 residues. Phosphorylated β-catenin is ubiquitinated to be degraded by proteasome [23–25]. Expression levels of p-β-catenin (S33/S37/T41), p-β-catenin (T41/S45), and p-β-catenin (S33/S37) were reduced in AQP1/MDA-MB-231 cells. We further examined several molecules involved in the regulation of β-catenin degradation by immunoblot analysis. However, we found no significant changes in the expression of the major components of degradation complex, including low-density-lipoprotein-related protein 6 (LRP6), Axin1, and GSK3β. We also examined the expression of p-GSK3β (S9; the inactivated form of GSK3β) and p-GSK3β (Y216; the activated form of GSK3β) and did not find any significant difference (Fig. 4f and Supplementary Fig. S5d). Consequently, overexpression of AQP1 inhibited the degradation of β-catenin via the ubiquitin-proteasome system (UPS), without inhibiting the expression and activity of degradation complex.

The C-terminal tail of AQP1 was required for the effects of AQP1 on migration and proliferation in PASMC [26]. Then, lentivirus-expressing Flag-labeled AQP1 fragments were transfected into MDA-MB-231 cells, which were designated as Flag-AQP1-ΔCT/MDA-MB-231, Flag-AQP1-CT/MDA-MB-231, and Flag-AQP1-6×Helix-CT/MDA-MB-231, respectively. And, the expression was detected by western blot (Supplementary Fig. S5e). Afterwards, Co-IP experiments showed that the C-terminal tail of AQP1 interacted with β-catenin in Flag-AQP1-CT/MDA-MB-231 cells (Fig. 4g). Immunofluorescence analysis showed similar results (Supplementary Fig. S5f, g). Subsequently, we measured the half-life of β-catenin following inhibition of new protein synthesis by CHX. The half-life was reduced in AQP1-ΔCT/MDA-MB-231 cells and increased in AQP1-CT/MDA-MB-231 cells (Supplementary Fig. S5h). Next, we discovered that the level of ubiquitination of β-catenin was upregulated in AQP1-ΔCT/MDA-MB-231 cells (Fig. 4h). We further examined several molecules involved in the regulation of β-catenin degradation by immunoblot analysis in AQP1/MDA-MB-231 cells and AQP1-ΔCT/MDA-MB-231 cells. It was found that β-catenin expression significantly decreased, while expression of p-β-catenin (S33/S37/T41), p-β-catenin (T41/S45), and p-β-catenin (S33/S37) was increased in AQP1-ΔCT/MDA-MB-231 cells. We found no significant changes in the expression of LRP6, Axin1, GSK3β, p-GSK3β (S9), and p-GSK3β (Y216) as well (Fig. 4i and Supplementary Fig. S5i).

β-catenin consists of a NH2-terminal domain, 12 armadillo repeats (12 × arm), and a COOH-terminal domain. The lentivirus-expressing Flag-labeled β-catenin fragments were transfected into mGFP-AQP1/HEK-293T cells, the expression was detected by western blot (Supplementary Fig. S5j). Subsequently, Co-IP experiments showed that it was the 12 armadillo repeats (12 × arm) of β-catenin that specifically interacted with AQP1 (Fig. 4j). Then these β-catenin fragments were transfected into HEK-293T cells, the expression was verified by western blot (Supplementary Fig. S5k). Additionally, Co-IP experiments also showed that the 12 armadillo repeats (12 × arm) of β-catenin specifically interacted with GSK3β (Fig. 4k). Next, we performed immunoprecipitation experiments and found that overexpression of AQP1 impaired the interaction between β-catenin and GSK3β (Fig. 4l). And, it is worth mentioning that the interaction between β-catenin and GSK3β was recovered when AQP1 lacked the C-terminal tail (Fig. 4m). Therefore, AQP1 and GSK3β competitively interacted with the 12 armadillo repeats (12 × arm) of β-catenin and then inhibited the degradation of β-catenin. Furthermore, we found that AQP1-ΔCT/MDA-MB-231 cells were less sensitive to EPI than AQP1/MDA-MB-231 cells (Fig. 4n and Supplementary Fig. S5l). Therefore, the C-terminal tail was necessary for AQP1 to upregulate the sensitivity to EPI by suppressing the ubiquitin-proteasome degradation pathway of β-catenin.

### AQP1-induced β-catenin nuclear translocation did not activate Wnt downstream target genes

**Fig. 5:**
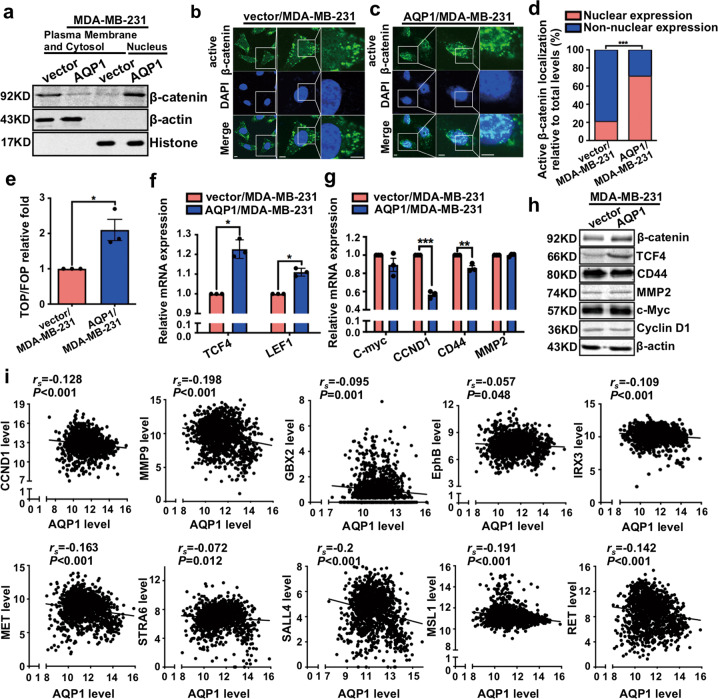
AQP1-induced β-catenin nuclear translocation did not activate Wnt downstream target genes. **a** Western blot analysis of β-catenin expression in plasma membrane, cytosol, and nucleus of breast cancer cells. β-actin and histone were used as specific markers for cytoplasm and nuclei, respectively. **b**, **c** Immunofluorescence analysis showed the localization of active β-catenin in vector/MDA-MB-231 (**b**) and AQP1/MDA-MB-231 (**c**) cells. Insets showed a high-magnification view of the indicated region. Scale bars: 50 μm. **d** Ratio of nuclear expression of active β-catenin was shown in a graph representation (*χ*^2^ test, ***P < 0.001). **e** Overexpression of AQP1 activated TOP-Flash reporter by TOP/FOP Flash experiments. Values were expressed as mean ± SEM (two-tailed Student’s *t* test, *P < 0.05). **f** Relative mRNA levels of *TCF4* and *LEF1* were quantified by RT-qPCR. Values were expressed as mean ± SEM (two-tailed Student’s *t* test, *P < 0.05). **g** Relative mRNA levels of *C-myc*, *CCND1*, *CD44*, and *MMP2* were quantified by RT-qPCR. Values were expressed as mean ± SEM (two-tailed Student’s *t* test, **P < 0.01, ***P < 0.001). **h** Western blot for the indicated proteins were performed. β-actin was the loading control. **i** The 1218 breast cancer patients’ RNAseq data in TCGA was analyzed. *AQP1* was negatively associated with Wnt downstream target genes, such as *CCND1*, *MMP9, GBX2*, *EphB*, *IRX3*, *MET*, *STRA6*, *SALL4*, *MSL1*, and *RET*. *P* value was calculated by Spearman’s Rank-Correlation test. Experiments **a**–**h** were independently repeated for three times.

Our cytosol/nuclear separation experiments demonstrated that AQP1 overexpression promoted β-catenin nuclear import (Fig. 5a). The immunofluorescence also showed, high expression of AQP1 promoted nuclear localization of active β-catenin (Fig. 5b–d). Supplementary Figure S6a–c showed that high expression of AQP1 promoted nuclear localization of β-catenin. It was well established that nuclear β-catenin associated with members of TCF/LEF family to mediate the expression of Wnt target genes. Therefore, we applied the TOP/FOP Flash reporter to ascertain whether AQP1 affects β-catenin-TCF/LEF transcriptional activity [25]. Overexpression of AQP1 activated TOP-Flash reporter and increased mRNA levels of *TCF4* and *LEF1* (Fig. 5e, f). When we examined the transcriptional activity of Wnt downstream target genes, such as *C-myc, CCND1, CD44*, and *MMP2*, they were not increased in AQP1/MDA-MB-231 cells (Fig. 5g). Western blot confirmed these results (Fig. 5h). We also conducted a correlation analysis by using the 1218 breast cancer patients’ RNAseq data from TCGA. We found that *AQP1* was negatively correlated with Wnt downstream target genes, such as *CCND1, MMP9, GBX2, EphB, IRX3, MET, STRA6, SALL4, MSL1*, and *RET* (Fig. 5i). Together, these results strongly supported that AQP1-induced β-catenin nuclear translocation did not activate Wnt downstream target genes.

### Nuclear β-catenin interacted with TopoIIα and enhanced its activity, thereby upregulating chemosensitivity to EPI

**Fig. 6:**
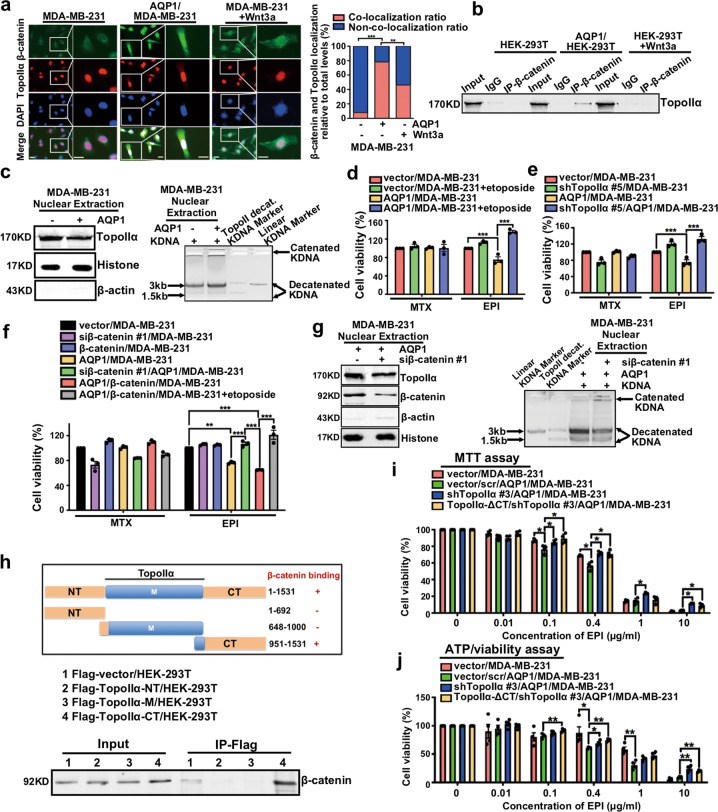
Nuclear β-catenin interacted with TopoIIα and enhanced its activity, thereby upregulating chemosensitivity to EPI. **a** Immunofluorescence analysis showed the localization of β-catenin and TopoIIα in MDA-MB-231, AQP1/MDA-MB-231, and MDA-MB-231 cells treated with Wnt3a (50 ng/ml) for 24 h (left panel). Insets showed a high-magnification view of the indicated region. Scale bars: 100 μm. The fluorescence intensity in a graph representation and values were expressed as mean ± SEM (*χ*^2^ test, **P < 0.01, ***P < 0.001; right panel). **b** Co-immunoprecipitation with β-catenin antibody in HEK-293T, AQP1/HEK-293T, and HEK-293T+Wnt3a cells showed the interaction of β-catenin with TopoIIα. **c** The expression (left panel) and catalytic activity (right panel) of TopoIIα were detected in vector/MDA-MB-231 cells and AQP1/MDA-MB-231 cells. **d** After TopoIIα activity inhibitor etoposide (0.1 μg/ml) treatment for 6 h, vector/MDA-MB-231 and AQP1/MDA-MB-231 cells were treated with EPI (0.5276 μg/ml) or MTX (0.01347 mg/ml) for 48 h, and cell viability was tested by MTT assay. Cell viability of vector/MDA-MB-231 was normalized to 100%. Values were expressed as mean ± SEM (two-tailed Student’s *t* test, ***P < 0.001). **e** Vector/MDA-MB-231, shTopoIIα/MDA-MB-231, AQP1/MDA-MB-231, and shTopoIIα/AQP1/MDA-MB-231 cells were treated with EPI (0.5276 μg/ml) or MTX (0.01347 mg/ml) for 48 h, and cell viability was tested by MTT assay. Cell viability of vector/MDA-MB-231 was normalized to 100%. Values were expressed as mean ± SEM (two-tailed Student’s *t* test, ***P < 0.001). **f** Cell viability of vector/MDA-MB-231, siβ-catenin/MDA-MB-231, β-catenin/MDA-MB-231, AQP1/MDA-MB-231, siβ-catenin/AQP1/MDA-MB-231, AQP1/β-catenin/MDA-MB-231, and AQP1/β-catenin/MDA-MB-231+etoposide with EPI (0.5276 μg/ml) or MTX (0.01347 mg/ml) treatment for 48 h was tested by MTT assay. Cell viability of vector/MDA-MB-231 was normalized to 100%. Values were expressed as mean ± SEM (two-tailed Student’s *t* test, **P < 0.01, ***P < 0.001). **g** The expression (left panel) and catalytic activity (right panel) of TopoIIα were tested in AQP1/MDA-MB-231 cells and siβ-catenin/AQP1/MDA-MB-231 cells. **h** A schematic illustration of TopoIIα and TopoIIα-CT domain mediated the interaction with β-catenin by co-immunoprecipitation experiments. **i**, **j** Cell viability of vector**/**MDA-MB-231, vector/scr/AQP1/MDA-MB-231, shTopoIIα/AQP1/MDA-MB-231, and TopoIIα-ΔCT/shTopoIIα/AQP1/MDA-MB-231 with different concentration of EPI treatment for 48 h was tested by MTT (**i**) or ATP/viability (**j**) assay. Values were expressed as mean ± SEM (two-tailed Student’s *t* test, *P < 0.05, **P < 0.01). All experiments were independently repeated for three times.

Recent studies had provided sufficient evidence that β-catenin interacted with TopoIIα and served as a novel transcriptional co-activator in colorectal cancer [27]. We verified the interaction between β-catenin and TopoIIα in MDA-MB-231, AQP1/MDA-MB-231, and MDA-MB-231+Wnt3a cells. The immunofluorescence experiments showed that β-catenin and TopoIIα had significant co-localization in AQP1/MDA-MB-231 cells (Fig. 6a), and Co-IP experiments revealed similar results (Fig. 6b). We further evaluated the correlation between the expression of β-catenin and TopoIIα in a cohort of 70 IDC patients. However, there was no significant correlation (Supplementary Table S8), considering that TopoIIα localized in the nucleus, and β-catenin was mainly expressed in the cytoplasm and membrane of breast cancer cells.

TopoIIα is a well-recognized indicator of the ability of cell proliferation, but as one of the targets of anthracyclines, its catalytic activity associates with the sensitivity of anthracyclines [4, 8]. We examined the expression and catalytic activity of TopoIIα by immunoblot assay and TopoGen decatenation assay and found that overexpression of AQP1 upregulated the activity of TopoIIα but had no significant effect on TopoIIα expression (Fig. 6c). Next, we applied a TopoIIα activity inhibitor etoposide and found that AQP1/MDA-MB-231 cells with etoposide treatment were less sensitive to EPI compared with AQP1/MDA-MB-231 cells (Fig. 6d). In addition, the TopoIIα expression was knocked down by five different shRNAs in MDA-MB-231 cells and AQP1/MDA-MB-231 cells, respectively (Supplementary Fig. S6d, e). We also found that shTopoIIα/AQP1/MDA-MB-231 cells were less sensitive to EPI than AQP1/MDA-MB-231 cells (Fig. 6e). Thus, overexpression of AQP1 participated in the upregulation of EPI sensitivity by enhancing the activity of TopoIIα.

To further verify whether AQP1 modulates TopoIIα activity and EPI sensitivity by regulating β-catenin, we knocked down β-catenin expression and overexpressed mGFP-HA-β-catenin in MDA-MB-231 and AQP1/MDA-MB-231 cells, respectively (Supplementary Fig. S6f–i). Knocked down β-catenin reduced the sensitivity of AQP1/MDA-MB-231 cells to EPI, and inhibiting the activity of TopoIIα reduced the sensitivity of AQP1/β-catenin/MDA-MB-231 cells to EPI (Fig. 6f). Then, we compared the activity of TopoIIα in AQP1/MDA-MB-231 cells and siβ-catenin/AQP1/MDA-MB-231 cells and found that knocking down β-catenin inhibited the activity of TopoIIα (Fig. 6g).

To determine the region of TopoIIα necessary for the interaction of TopoIIα with β-catenin, serial deletion mutants of TopoIIα were constructed and transfected into HEK-293T cells, and Co-IP experiments confirmed the C-terminal tail of TopoIIα interacted with β-catenin (Fig. 6h and Supplementary Fig. S6j). Next, we constructed TopoIIα-ΔCT/shTopoIIα/AQP1/MDA-MB-231 cells, which were applied to disrupt the interaction between β-catenin and TopoIIα (Supplementary Fig. S6k). MTT assay and ATP/viability assay revealed that AQP1/MDA-MB-231 cells with TopoIIα lacking a C-terminal tail had inhibited EPI sensitivity (Fig. 6i, j). Therefore, the C-terminal tail of TopoIIα was necessary for β-catenin to regulate EPI sensitivity in AQP1/MDA-MB-231 cells. Above results showed that AQP1 promoted β-catenin nuclear translocation, and nuclear β-catenin interacted with TopoIIα and enhanced TopoIIα’s activity, thereby upregulating EPI chemosensitivity.

### miR-320a-3p attenuates EPI chemosensitivity by inhibiting AQP1 expression in breast cancer

**Fig. 7:**
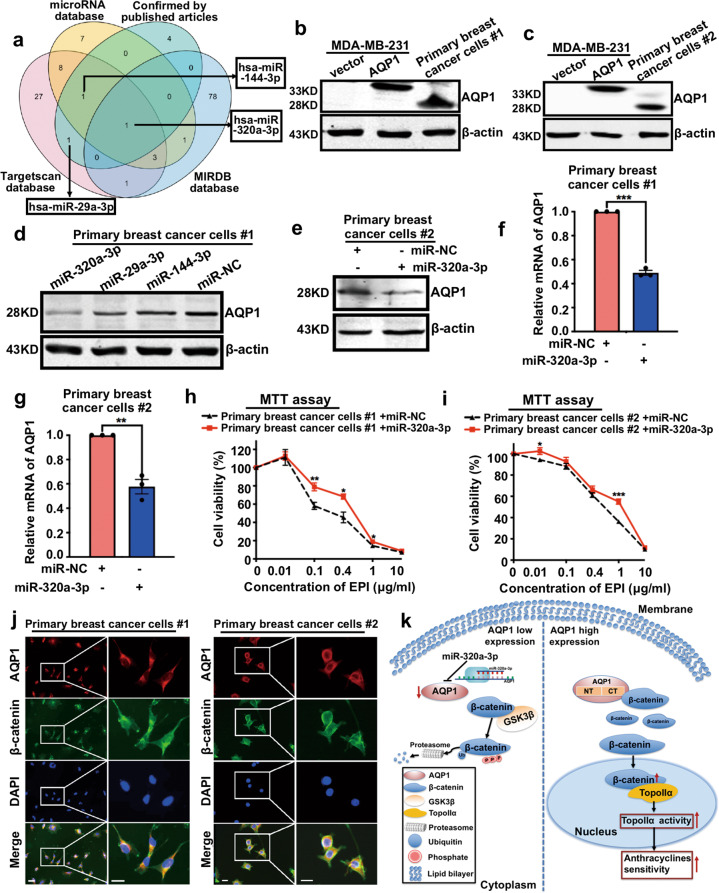
miR-320a-3p attenuates EPI chemosensitivity by inhibiting AQP1 expression in breast cancer. **a** A Venn diagram showed several candidate microRNAs potentially regulating AQP1 based on a combination analysis of three public databases and verified data retrieved from published literature. **b**, **c** Western blot of AQP1 expression in primary breast cancer cells #1 (**b**) and #2 (**c**), vector/MDA-MB-231 and AQP1/MDA-MB-231 cells. β-actin was the loading control. **d**, **e** AQP1 expression was detected in primary breast cancer cells #1 (**d**) and #2 (**e**) transfected with microRNAs by western blot analysis. β-actin was the loading control. **f**, **g** The mRNA level of AQP1 was detected in miR-NC/primary breast cancer cells and miR-320a-3p/primary breast cancer cells by RT-qPCR. GAPDH was as control. (two-tailed Student’s *t* test, **P < 0.01, ***P < 0.001). **h**, **i** The viability of miR-NC/primary breast cancer cells and miR-320a-3p/primary breast cancer cells with different concentration of EPI treatment for 48 h was detected by MTT assay. Values were expressed as mean ± SEM (two-tailed Student’s *t* test, *P < 0.05, **P < 0.01, ***P < 0.001). **j** Co-localization of AQP1 and β-catenin in primary breast cancer cells. Insets showed a high-magnification view of the indicated region. Scale bars: 100 μm. **k** The signaling pathways of miR-320a-3p/AQP1 axis function in anthracycline chemotherapy. Experiments **b**–**j** were independently repeated for three times.

After demonstrating the relationship between AQP1 and anthracycline chemotherapy, we intended to apply miRNAs to regulate the expression of AQP1, thereby regulating the sensitivity of breast cancer cells to anthracyclines. Combination analyses of three public databases (TargetScan, miRanda, and miPDB) and the verified data in published articles suggested several miRNAs potentially regulated AQP1, including miR-320a-3p (Fig. 7a). We downloaded the miRNA expression profile and doxorubicin sensitivity (IC_50_) data of breast cancer cell lines (*n* = 44) from CCLE. We then divided breast cancer cell lines (*n* = 44) into the miR-320a-3p high group and the miR-320a-3p low group by using the median expression level as cutoff point. We found that miR-320a-3p expression was significantly negatively correlated with anthracyclines sensitivity (Supplementary Fig. S7a). Next, we downloaded the miRNA and mRNA gene expression profiles of breast cancer tissues (*n* = 98) from GEO (ID: GSE19783). The data showed that AQP1 expression was significantly negatively correlated with miR-320a-3p level in breast cancer, especially in the luminal subtype (Supplementary Fig. S7b, c). Then we applied primary breast cancer cells from two patients and found that overexpression of miR-320a-3p reduced the expression of AQP1 (Fig. 7b–e). The mRNA level of AQP1 was detected by RT-qPCR, and decreased expression of AQP1 was observed in miR-320a-3p/primary breast cancer cells (Fig. 7f, g). Furthermore, we found that overexpression of miR-320a-3p attenuated the sensitivity of primary breast cancer cells to EPI (Fig. 7h, i). The co-localization of AQP1 and β-catenin was confirmed in primary breast cancer cells by immunofluorescence analysis (Fig. 7j). Finally, we summarized the signaling pathways in Fig. 7k to outline our hypothesis in which the miR-320a-3p/AQP1 axis promotes the sensitivity of anthracycline chemotherapy in breast cancer.

## Discussion

AQP1 was originally identified as a small hydrophobic integral transmembrane protein that mainly functions in trans-cellular water transport in response to osmotic gradients [28–30]. Over the past decade, increasing evidence demonstrated new functions of AQP1: through interactions with specific proteins, it can participate in the progression of multiple cancers [31–36]. AQP1 can interact with Lin7/β-catenin or FAK and regulate cancer cell migration [37–39]. Our previous study also revealed that AQP1 localized predominantly in the cytoplasm of 95% (324/341) IDC patients, and the high cytoplasmic expression of AQP1 indicated a worse prognosis outcome [3]. The expression pattern of AQP1 in breast cancer cells suggests a possible relation between its cytoplasm localization and its function in breast cancer development.

Our present study showed, through the ubiquitin-proteasome system in AQP1 overexpressed breast cancer cells, the AQP1 overexpression inhibited the degradation of β-catenin, thus promoting β-catenin accumulation in the cytoplasm. It is known that in the classical β-catenin degradation mechanism, the cytosolic β-catenin is efficiently captured and phosphorylated by the destruction complexes such as GSK3β. The phosphorylated β-catenin can be ubiquitinated by the β-TrCP ubiquitin E3 ligase to be degraded by the proteasome [24, 25, 40]. Additional proteins were identified to be involved in the β-catenin degradation process. The transcription factor specificity protein 1 (SP1) and Yes-associated protein (YAP) can individually interact with β-catenin and both SP1 and YAP are part of the destruction complex to regulate the β-catenin stability in cancer cells [41–43]. The above evidence illustrates a complex mechanism of β-catenin degradation whose details remain to be further explored. Our study here suggests a new role of AQP1 that acts as a possible regulatory component in the β-catenin degradation pathway.

As previously established, the cytoplasmic accumulation of β-catenin induced by membranous Wnt ligands stimulation could lead to β-catenin’s translocation into nucleus where the β-catenin interacted with several transcriptional co-factors such as TCF/LEF to activate the Wnt transcriptional program [44, 45]. However, our present study showed that the cytoplasmic accumulation and nuclear localization of β-catenin induced by the overexpressed AQP1 in breast cancer did not activate the Wnt downstream target genes such as *C-Myc, CCND1, CD44*, and *MMP2*. We also demonstrated that AQP1 and GSK3β could competitively interact with the 12 armadillo repeats of β-catenin in the inhibition of the ubiquitin-proteasome degradation pathway of β-catenin. It is plausible that this competitive interaction alters the spatial conformation or post-translational modification of the β-catenin, which may differ from that in the Wnt-stimulated pathway.

In this study, we also found that miR-320a-3p inhibited AQP1 expression. Previous reports have demonstrated that miR-320 could directly target AQP1 3′-UTR and negatively regulate its expression, which was consistent with our results [46, 47]. Furthermore, we also revealed, for the first time, that miR-320a-3p could inhibit AQP1 expression to attenuate the EPI chemosensitivity.

In conclusion, our study here suggests the usage of AQP1 as a response predictor in the anthracycline chemotherapy. The application of our study includes, but is not limited to, facilitating screening of the most appropriate breast cancer patients (who have a high AQP1 expression) for better anthracycline chemotherapy and improved prognosis purposes.

## Supplementary information

Supplementary Fig. S1

Supplementary Fig. S2

Supplementary Fig. S3

Supplementary Fig. S4

Supplementary Fig. S5

Supplementary Fig. S6

Supplementary Fig. S7

Supplementary Figure legends

Supplemetary Table S1

Supplemetary Table S2

Supplemetary Table S3

Supplemetary Table S4

Supplemetary Table S5

Supplemetary Table S6

Supplemetary Table S7

Supplemetary Table S8

## References

[1] Harbeck N, Gnant M (2017). Breast cancer. Lancet.

[2] Peto R, Davies C, Godwin J, Gray R, Pan HC, Early Breast Cancer Trialists’ Collaborative G (2012). Comparisons between different polychemotherapy regimens for early breast cancer: meta-analyses of long-term outcome among 100,000 women in 123 randomised trials. Lancet.

[3] Qin F, Zhang H, Shao Y, Liu X, Yang L, Huang Y (2016). Expression of aquaporin1, a water channel protein, in cytoplasm is negatively correlated with prognosis of breast cancer patients. Oncotarget.

[4] Mordente A, Meucci E, Martorana GE, Tavian D, Silvestrini A (2017). Topoisomerases and anthracyclines: recent advances and perspectives in anticancer therapy and prevention of cardiotoxicity. Curr Med Chem.

[5] Marinello J, Delcuratolo M, Capranico G. Anthracyclines as topoisomerase II poisons: from early studies to new perspectives. Int J Mol Sci 2018;19:3480. 10.3390/ijms19113480 PMC6275052 30404148

[6] Li XJ, Zha QB, Ren ZJ, Tang JH, Yao YF (2015). Mechanisms of breast cancer resistance to anthracyclines or taxanes: an overview of the proposed roles of noncoding RNA. Curr Opin Oncol.

[7] Capeloa T, Benyahia Z, Zampieri LX, Blackman M, Sonveauz P (2020). Metabolic and non-metabolic pathways that control cancer resistance to anthracyclines. Semin Cell Dev Biol..

[8] Nitiss JL (2009). Targeting DNA topoisomerase II in cancer chemotherapy. Nat Rev Cancer.

[9] Jasra S, Anampa J (2018). Anthracycline use for early stage breast cancer in the modern era: a review. Curr Treat Options Oncol.

[10] Abiko T, Kawamura M, Izumi Y, Oyama T, Saito Y, Kobayashi K (2007). Prediction of anti-tumour effect of thermochemotherapy with in vitro thermochemosensitivity testing for non-small cell lung cancer. Int J Hyperther.

[11] Kawamura M, Gika M, Abiko T, Inoue Y, Oyama T, Izumi Y (2007). Clinical evaluation of chemosensitivity testing for patients with unresectable non-small cell lung cancer (NSCLC) using collagen gel droplet embedded culture drug sensitivity test (CD-DST). Cancer Chemother Pharmacol.

[12] Chen H, Yang M, Wang Q, Song F, Li X, Chen K (2019). The new identified biomarkers determine sensitivity to immune check-point blockade therapies in melanoma. Oncoimmunology.

[13] Gu F, Wang L, He J, Liu X, Zhang H, Li W (2014). Girdin, an actin-binding protein, is critical for migration, adhesion, and invasion of human glioblastoma cells. J Neurochem.

[14] Dai K, Qin F, Zhang H, Liu X, Guo C, Zhang M (2016). Low expression of BMPRIB indicates poor prognosis of breast cancer and is insensitive to taxane-anthracycline chemotherapy. Oncotarget.

[15] Zhang H, Yu F, Qin F, Shao Y, Chong W, Guo Z (2018). Combination of cytoplasmic and nuclear girdin expression is an independent prognosis factor of breast cancer. FASEB J.

[16] Shao Y, Chong W, Liu X, Xu Y, Zhang H, Xu Q (2019). Alternative splicing-derived intersectin1-L and intersectin1-S exert opposite function in glioma progression. Cell Death Dis.

[17] Wang Q, Ma S, Song N, Li X, Liu L, Yang S (2016). Stabilization of histone demethylase PHF8 by USP7 promotes breast carcinogenesis. J Clin Invest.

[18] Gu F, Zhang H, Qin F, Liu X, Li W, Fu L (2015). Intersectin1-S, a multidomain adapter protein, is essential for malignant glioma proliferation. Glia.

[19] Bower JJ, Karaca GF, Zhou Y, Simpson DA, Cordeiro-Stone M, Kaufmann WK (2010). Topoisomerase IIalpha maintains genomic stability through decatenation G(2) checkpoint signaling. Oncogene.

[20] Gardner L, Malik R, Shimizu Y, Mullins N, ElShamy WM (2011). Geminin overexpression prevents the completion of topoisomerase IIalpha chromosome decatenation, leading to aneuploidy in human mammary epithelial cells. Breast Cancer Res.

[21] Chen L, Guo P, He Y, Chen Z, Chen L, Luo Y (2018). HCC-derived exosomes elicit HCC progression and recurrence by epithelial-mesenchymal transition through MAPK/ERK signalling pathway. Cell Death Dis.

[22] van Noort M, Meeldijk J, van der Zee R, Destree O, Clevers H (2002). Wnt signaling controls the phosphorylation status of beta-catenin. J Biol Chem.

[23] Li VS, Ng SS, Boersema PJ, Low TY, Karthaus WR, Gerlach JP (2012). Wnt signaling through inhibition of beta-catenin degradation in an intact Axin1 complex. Cell.

[24] Lu L, Gao Y, Zhang Z, Cao Q, Zhang X, Zou J (2015). Kdm2a/b lysine demethylases regulate canonical Wnt signaling by modulating the stability of nuclear beta-catenin. Dev Cell.

[25] Zhang N, Wei P, Gong A, Chiu WT, Lee HT, Colman H (2011). FoxM1 promotes beta-catenin nuclear localization and controls Wnt target-gene expression and glioma tumorigenesis. Cancer Cell.

[26] Lai N, Lade J, Leggett K, Yun X, Baksh S, Chau E (2014). The aquaporin 1 C-terminal tail is required for migration and growth of pulmonary arterial myocytes. Am J Respir Cell Mol Biol.

[27] Huang L, Shitashige M, Satow R, Honda K, Ono M, Yun J (2007). Functional interaction of DNA topoisomerase IIalpha with the beta-catenin and T-cell factor-4 complex. Gastroenterology.

[28] Agre P, King LS, Yasui M, Guggino WB, Ottersen OP, Fujiyoshi Y (2002). Aquaporin water channels–from atomic structure to clinical medicine. J Physiol.

[29] Ribatti D, Ranieri G, Annese T, Nico B (2014). Aquaporins in cancer. Biochim Biophys Acta.

[30] Papadopoulos MC, Saadoun S (2015). Key roles of aquaporins in tumor biology. Biochim Biophys Acta.

[31] Papadopoulos MC, Saadoun S, Verkman AS (2008). Aquaporins and cell migration. Pflugers Arch.

[32] Leggett K, Maylor J, Undem C, Lai N, Lu W, Schweitzer K (2012). Hypoxia-induced migration in pulmonary arterial smooth muscle cells requires calcium-dependent upregulation of aquaporin 1. Am J Physiol Lung Cell Mol Physiol.

[33] Saadoun S, Papadopoulos MC, Hara-Chikuma M, Verkman AS (2005). Impairment of angiogenesis and cell migration by targeted aquaporin-1 gene disruption. Nature.

[34] Hu J, Verkman AS (2006). Increased migration and metastatic potential of tumor cells expressing aquaporin water channels. FASEB J.

[35] Hayashi S, Takahashi N, Kurata N, Yamaguchi A, Matsui H, Kato S (2009). Involvement of aquaporin-1 in gastric epithelial cell migration during wound repair. Biochem Biophys Res Commun.

[36] Gao L, Gao Y, Li X, Howell P, Kumar R, Su X (2012). Aquaporins mediate the chemoresistance of human melanoma cells to arsenite. Mol Oncol.

[37] La Porta C (2010). AQP1 is not only a water channel: It contributes to cell migration through Lin7/beta-catenin. Cell Adh Migr.

[38] Meng F, Rui Y, Xu L, Wan C, Jiang X, Li G (2014). Aqp1 enhances migration of bone marrow mesenchymal stem cells through regulation of FAK and beta-catenin. Stem Cells Dev.

[39] Yun X, Jiang H, Lai N, Wang J, Shimoda LA (2017). Aquaporin 1-mediated changes in pulmonary arterial smooth muscle cell migration and proliferation involve beta-catenin. Am J Physiol Lung Cell Mol Physiol.

[40] Kim SE, Huang H, Zhao M, Zhang X, Zhang A, Semonov MV (2013). Wnt stabilization of beta-catenin reveals principles for morphogen receptor-scaffold assemblies. Science.

[41] Azzolin L, Panciera T, Soligo S, Enzo E, Bicciato S, Dupont S (2014). YAP/TAZ incorporation in the beta-catenin destruction complex orchestrates the Wnt response. Cell.

[42] Imajo M, Miyatake K, Iimura A, Miyamoto A, Nishida E (2012). A molecular mechanism that links Hippo signalling to the inhibition of Wnt/beta-catenin signalling. EMBO J.

[43] Liu B, Ma H, Liu Q, Xiao Y, Pan S, Zhou H (2019). MiR-29b/Sp1/FUT4 axis modulates the malignancy of leukemia stem cells by regulating fucosylation via Wnt/beta-catenin pathway in acute myeloid leukemia. J Exp Clin Cancer Res.

[44] Behrens J, von Kries JP, Kuhl M, Bruhn L, Wedlich D, Grosschedl R (1996). Functional interaction of beta-catenin with the transcription factor LEF-1. Nature.

[45] Molenaar M, van de Wetering M, Oosterwegel M, Peterson-Maduro J, Godsave S, Korinek V (1996). XTcf-3 transcription factor mediates beta-catenin-induced axis formation in Xenopus embryos. Cell.

[46] Luo L, Yang R, Zhao S, Chen Y, Hong S, Wang K (2018). Decreased miR-320 expression is associated with breast cancer progression, cell migration, and invasiveness via targeting Aquaporin 1. Acta Biochim Biophys Sin.

[47] Wei WF, Zhou CF, Wu XG, He LN, Wu LF, Chen XJ (2017). MicroRNA-221-3p, a TWIST2 target, promotes cervical cancer metastasis by directly targeting THBS2. Cell Death Dis.

