# Supplementary figures and images for: Aquaporin 1 promotes sensitivity of anthracycline chemotherapy in breast cancer by inhibiting β-catenin degradation to enhance TopoIIα activity
Source: Cell Death Differ. 2020 Aug 19;28(1):382–400. doi: 10.1038/s41418-020-00607-9 (PMC7852611; doi:10.1038/s41418-020-00607-9)

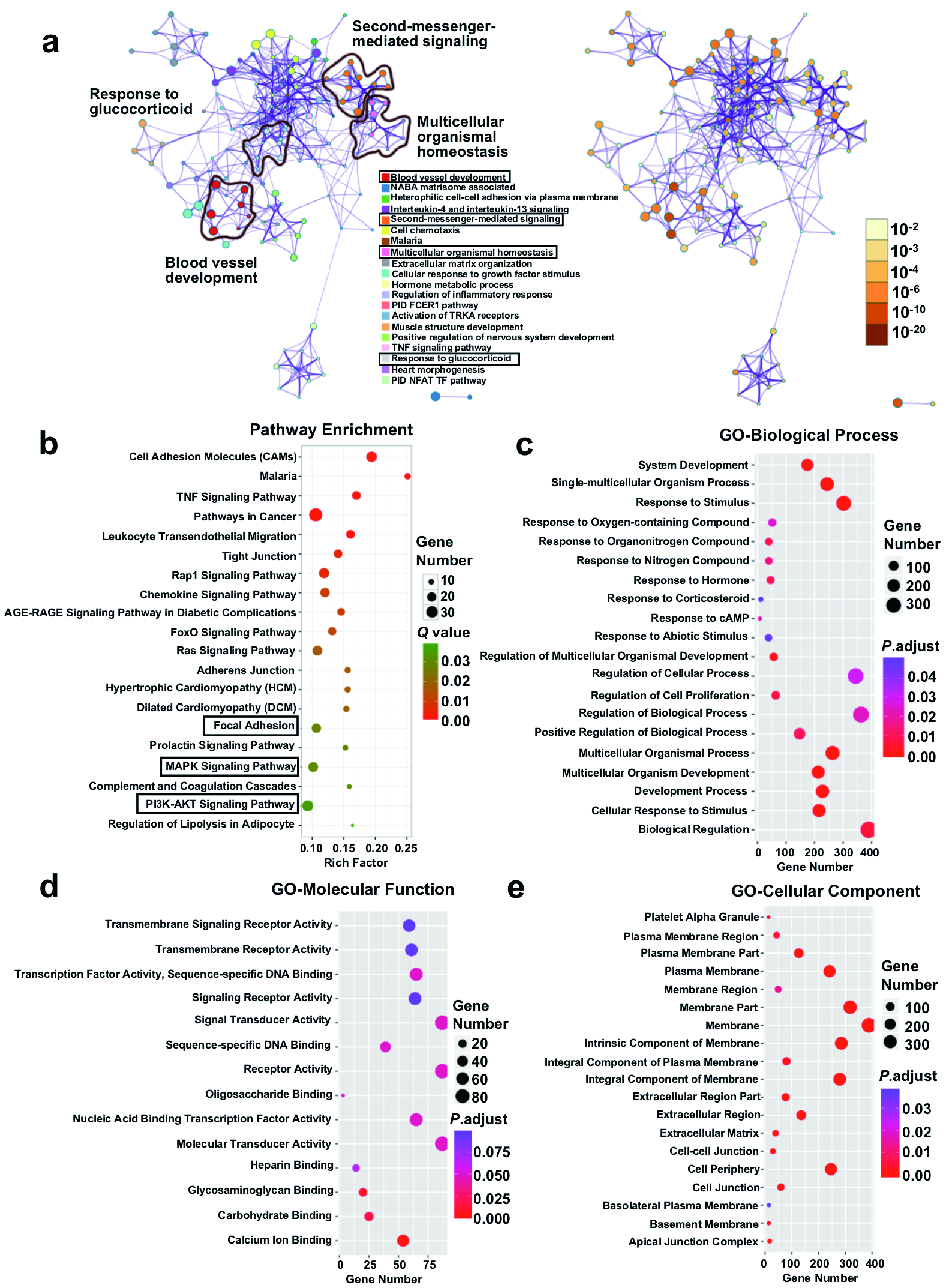

Supplement: Supplementary file 1 — Supplementary Fig. S1 [file 41418_2020_607_MOESM1_ESM.tif]

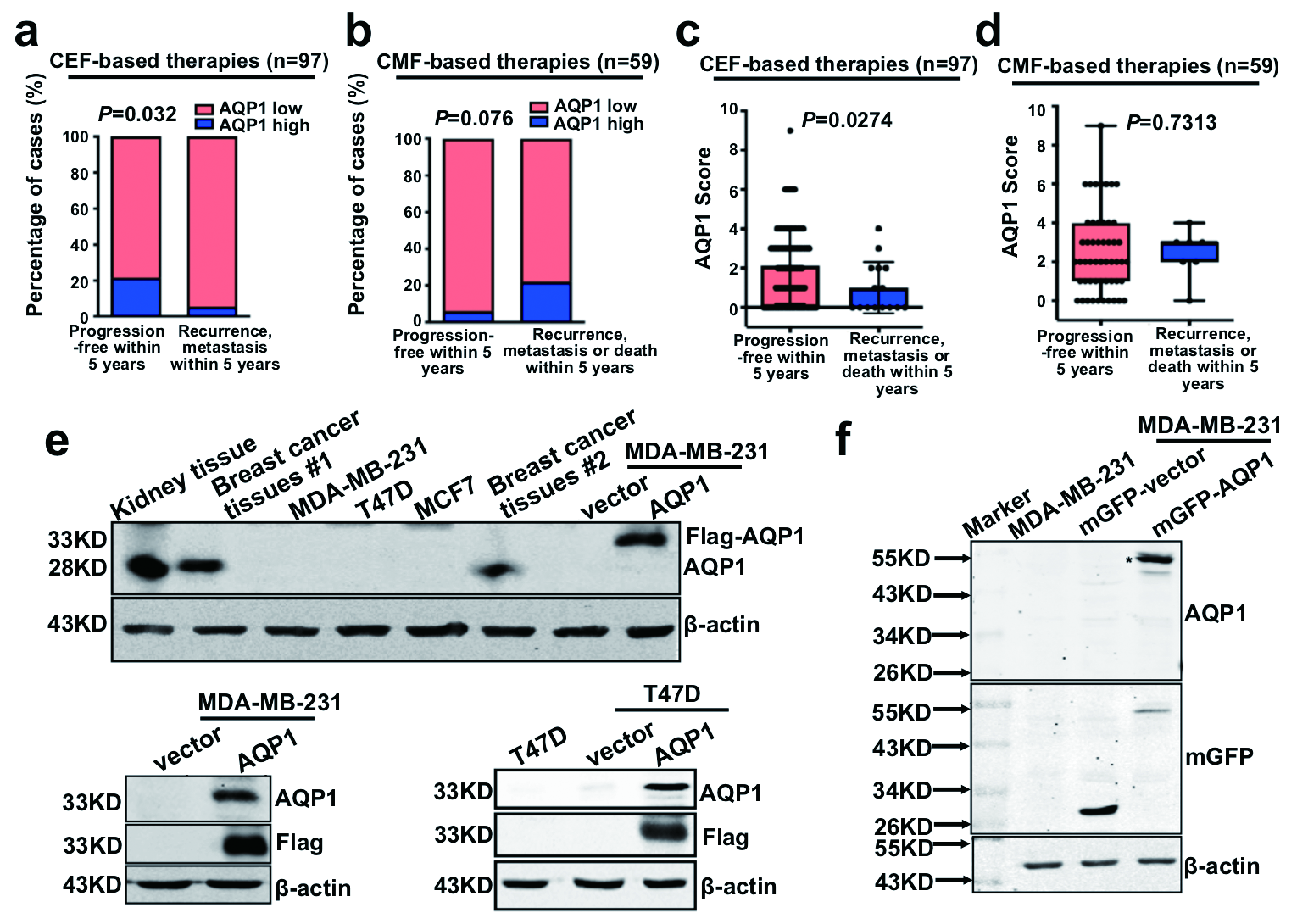

Supplement: Supplementary file 2 — Supplementary Fig. S2 [file 41418_2020_607_MOESM2_ESM.tif]

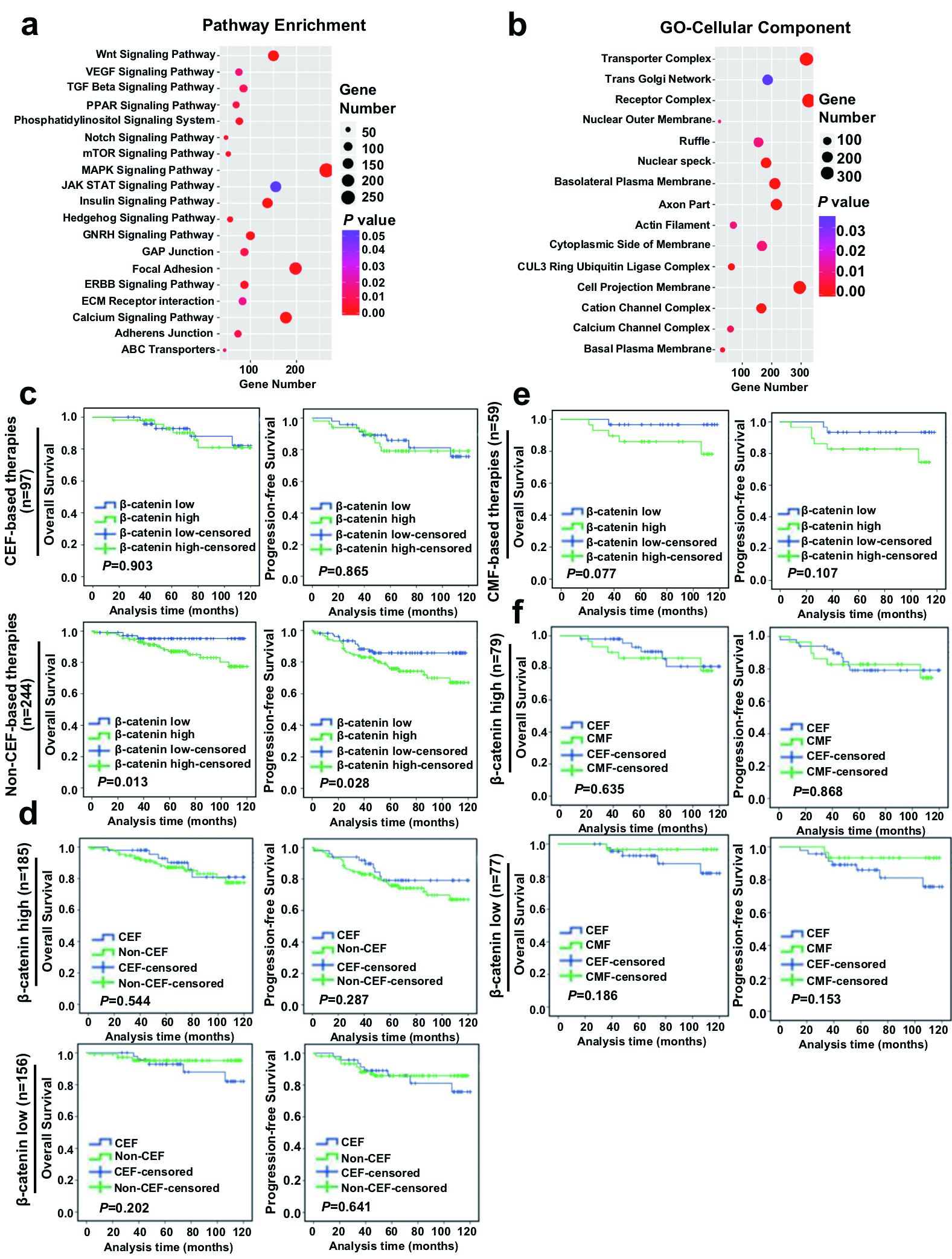

Supplement: Supplementary file 3 — Supplementary Fig. S3 [file 41418_2020_607_MOESM3_ESM.tif]

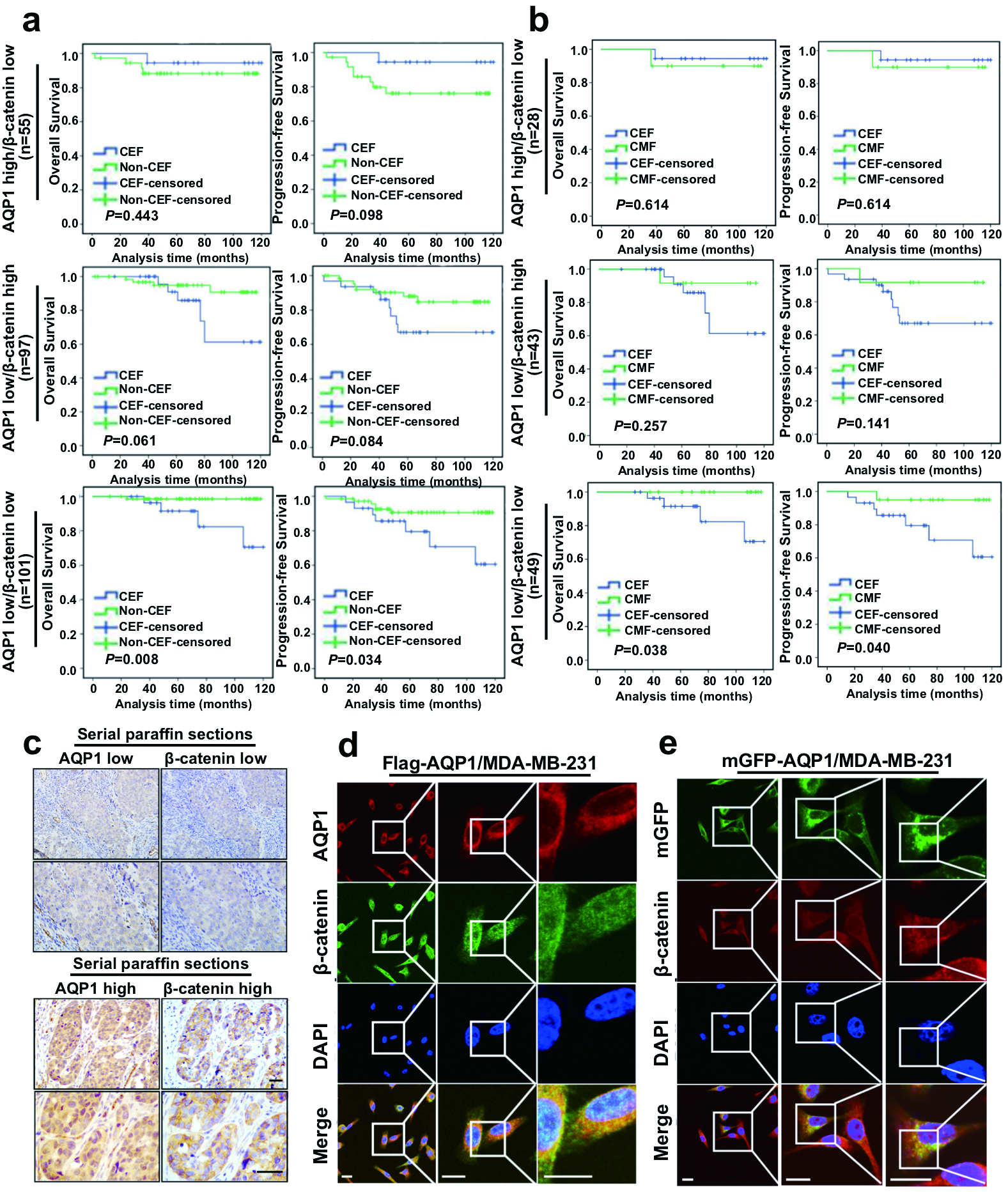

Supplement: Supplementary file 4 — Supplementary Fig. S4 [file 41418_2020_607_MOESM4_ESM.tif]

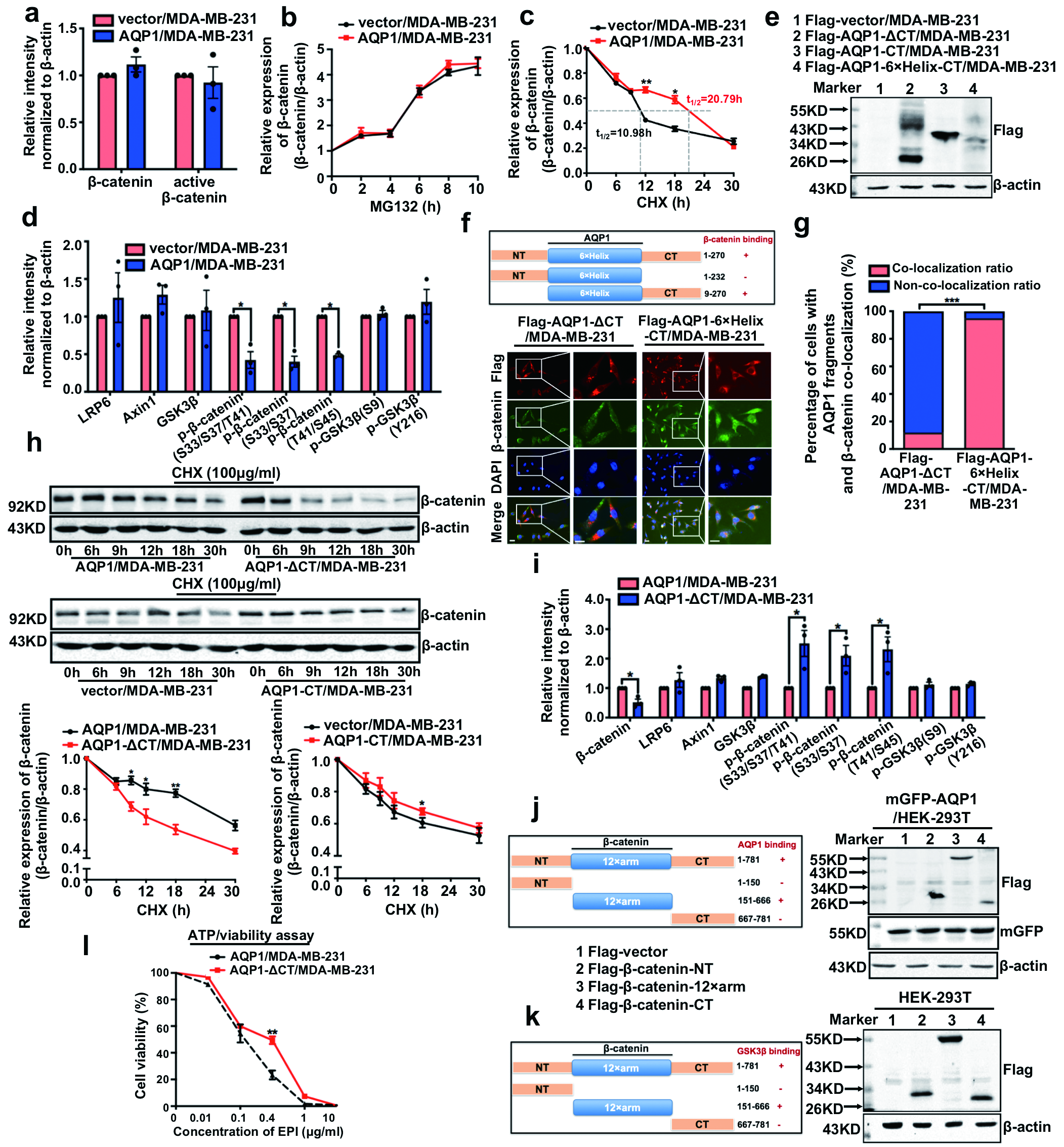

Supplement: Supplementary file 5 — Supplementary Fig. S5 [file 41418_2020_607_MOESM5_ESM.tif]

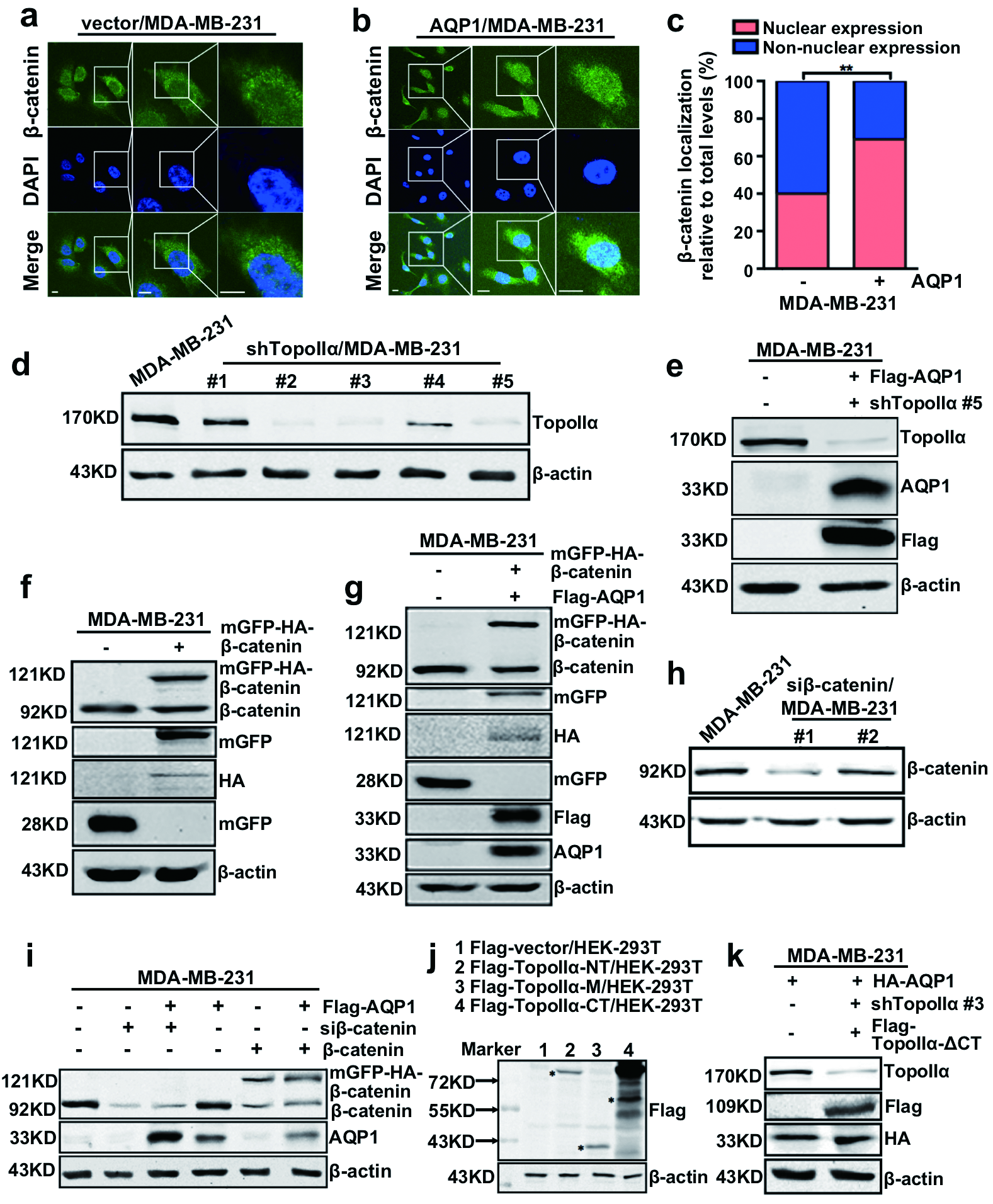

Supplement: Supplementary file 6 — Supplementary Fig. S6 [file 41418_2020_607_MOESM6_ESM.tif]

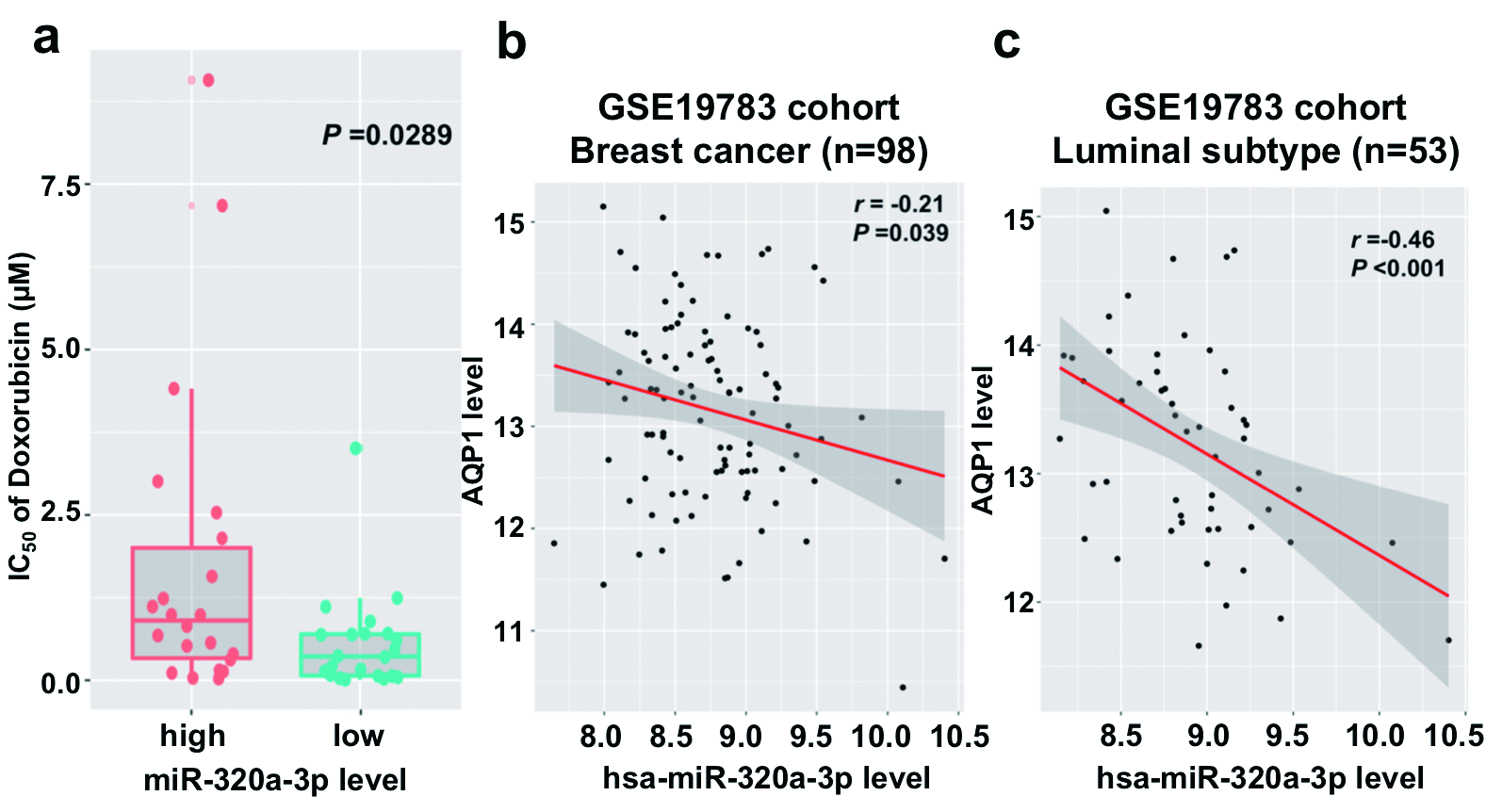

Supplement: Supplementary file 7 — Supplementary Fig. S7 [file 41418_2020_607_MOESM7_ESM.tif]
