# Supplementary material for: Aquaporin 1 promotes sensitivity of anthracycline chemotherapy in breast cancer by inhibiting β-catenin degradation to enhance TopoIIα activity
Source: Cell Death Differ. 2020 Aug 19;28(1):382–400. doi: 10.1038/s41418-020-00607-9 (PMC7852611; doi:10.1038/s41418-020-00607-9)
Supplement: Supplementary file 8 — Supplementary Figure legends [file 41418_2020_607_MOESM8_ESM.doc]

**Supplementary Figure legends**

**Supplementary Fig. S1. Gene enrichment analyses of differentially expressed genes (DEGs) in the EPI-sensitive group and the EPI-non-sensitive group.** **(a)** The metascape enrichment network visualization showed the intra-cluster and inter-cluster similarities of enriched terms, up to 20 terms per cluster. Cluster annotations were shown in color code. Network coloring on the right of the figure represented the *P* value of the enrichment pathways. **(b)** The rich factor plot of KEGG pathway enrichment analysis results. The degree of color represented the *Q* value. The size of node represented the gene number in this item. **(c-e)** GO functional annotation genes in the module obtained by GSEA. Y-axis showed the GO terms and x-axis showed the gene number of each term, the degree of color represented *P* value. (GO**:** Gene ontology, KEGG: Kyoto Encyclopedia of Genes and Genomes, GSEA: Gene Set Enrichment Analysis).

**Supplementary Fig. S2. High AQP1 expression indicated a favorable prognosis in breast cancer patients treated with CEF-based therapies. (a-b)** Among CEF-based therapies patients, 21.7% of patients who were progression-free within 5 years showed high AQP1 expression, while 5.4% of patients who developed recurrence or metastasis within 5 years exhibited high AQP1 expression (Chi-square test, *P*=0.032) **(a)**, but not in CMF-based therapies patients **(b)**. **(c-d)** Among CEF-based therapies patients, AQP1 expression score in patients who developed recurrence, metastasis, or death within 5 years was lower than those who were progression-free within 5 years (Mann-Whitney U test, *P*=0.0274) **(c)**, but not in CMF-based therapies patients **(d)**. **(e)** Western blot analyses of AQP1 expression in human breast cancer tissues and several breast cancer cell lines. AQP1 expression was detected by AQP1 and Flag antibodies in MDA-MB-231 and T47D cells. Mouse kidney tissues were used as a positive control and β-actin was the loading control. Experiments were independently repeated for three times. **(f)** Western blot analyses of mGFP-AQP1 expression in MDA-MB-231 cells. AQP1 expression was detected by AQP1 and mGFP antibodies. β-actin was the loading control. Experiments were independently repeated for three times.

**Supplementary Fig. S3. Differential gene expression analyses of 1218 breast cancer patients’ RNAseq data, and the expression of β-catenin was not associated with the prognosis of patients treated with anthracycline chemotherapy. (a)** The 1218 breast cancer patients’ RNAseq data was retrieved from TCGA. The gene expression profile was divided into two groups according to the expression of AQP1. The limma R package was applied to calculate the differentially expressed statistics. KEGG analysis of DEGs (|fold change|> 2, *P*<0.05). Y axis: KEGG category; X axis: Gene number; the degree of color denoted *P* value and the size of node represented the gene number in this item. **(b)** GO functional annotation genes in the module obtained by GSEA. Y-axis showed the GO terms, and x-axis showed the gene number of each term. The degree of color represented *P* value. **(c)** High expression of β-catenin suggested a shorter OS (left panel) and PFS (right panel) in patients treated with non-CEF-based chemotherapy (lower panel) than those treated with low β-catenin expression. In patients treated with CEF regimens (upper panel), no statistical difference was observed in OS (left panel) or PFS (right panel) between the high β-catenin expression group and the low β-catenin expression group (log-rank test). **(d)** Depending on the chemotherapy regimens, patients with high expression (upper panel) or low expression (lower panel) of β-catenin were divided into two groups. No significant difference was observed in OS (left panel) or PFS (right panel) between patients treated with CEF and non-CEF regimens. The expression of β-catenin was not associated with the prognosis of breast cancer patients treated with CEF-based chemotherapy (log-rank test). **(e)** The expression of β-catenin was not associated with the prognosis of breast cancer patients treated with CMF-based chemotherapy (log-rank test). **(f)** OS (left panel) and PFS (right panel) curves of patients with high expression (upper panel) of β-catenin or low expression (lower panel) of β-catenin who received CEF-based chemotherapy or CMF-based chemotherapy, respectively. The expression of β-catenin was not associated with the prognosis of breast cancer patients treated with anthracycline chemotherapy (log-rank test).

**Supplementary Fig. S4. AQP1 interacted with β-catenin in breast cancer, and analysis of the relationship between their expression and anthracyclines sensitivity.** **(a)** Patients were divided into four subgroups according to the expression of both AQP1 and β-catenin: AQP1 high/β-catenin high, AQP1 high/β-catenin low, AQP1 low/β-catenin high, AQP1 low/β-catenin low. The AQP1 low/β-catenin low subgroup patients who received CEF-based therapies had a shorter OS and PFS than patients receiving non-CEF regimens (log-rank test). **(b)** AQP1 low/β-catenin low subgroup patients who received CEF-based therapies had a shorter OS (left panel) and PFS (right panel) than CMF regimen patients (log-rank test). **(c)** The expression of AQP1 and β-catenin in IDC tissues was detected by immunohistochemistry analysis using serial paraffin sections and the expression of AQP1 was positively correlated with β-catenin expression. Scale bars: 100 μm. **(d)** Co-localization of AQP1 and β-catenin in Flag-AQP1/MDA-MB-231 cells. Insets showed a high-magnification view of the indicated region. Scale bars: 100 μm. **(e)** Co-localization of AQP1 and β-catenin in mGFP-AQP1/MDA-MB-231 cells. Insets showed a high-magnification view of the indicated region. Scale bars: 100 μm. Experiments **(d-e)** were independently repeated for three times.

**Supplementary Fig. S5. Quantitative analysis of Western blot bands intensity and the fluorescence localization. (a)** Expression of β-catenin and active β-catenin were quantitated by densitometry and normalized to β-actin expression. The bands intensity in a graph representation and values were expressed as mean ± SEM, representing the protein expression of the Western blot in Fig. 4a. Results were analyzed using two-tailed Student’s t test. **(b-c)** Quantitation was performed with β-actin as a normalizer, representing the protein expression of the Western blot in Fig. 4c-d. Values were expressed as mean ± SEM (two-tailed Student’s t test, **P*<0.05, ***P*<0.01). **(d)** Expression of indicated protein was quantitated by densitometry and normalized to β-actin expression. The bands intensity in a graph representation and values were expressed as mean ± SEM, representing the protein expression of the Western blot in Fig. 4f (two-tailed Student’s t test, **P*<0.01). **(e)** AQP1 deletion mutants were overexpressed in MDA-MB-231 cells and whole-cell lysates were directly subjected to Western blot using Flag antibody, β-actin was the loading control. **(f)** A schematic illustration of AQP1 and AQP1-CT domain mediated the interaction of AQP1 with β-catenin. Co-localization of Flag and β-catenin in Flag-AQP1-ΔCT/MDA-MB-231 (left panel) and Flag-AQP1-6×Helix-CT/MDA-MB-231 cells (right panel). Insets showed a high-magnification view of the indicated region. Scale bars: 100 μm. **(g)** Ratio of Flag and β-catenin co-localization in Flag-AQP1-ΔCT/MDA-MB-231 and Flag-AQP1-6×Helix-CT/MDA-MB-231 cells (Mann-Whitney U test, ****P*<0.001). **(h)** AQP1/MDA-MB-231, AQP1-ΔCT/MDA-MB-231, vector/MDA-MB-231 and AQP1-CT/MDA-MB-231 cells were treated with 100 μg/ml CHX, respectively, and harvested at the noted time points, followed by Western blot analysis. β-actin was the loading control (upper panel). Quantitation was performed with β-actin as a normalizer, representing the protein expression of the Western blot (lower panel). Values were expressed as mean ± SEM (two-tailed Student’s t test, **P*<0.05, ***P*<0.01). **(i)** Expression of indicated protein was quantitated by densitometry and normalized to the β-actin expression. The bands intensity in a graph representation and values were expressed as mean ± SEM, representing the protein expression of the Western blot in Fig. 4i (two-tailed Student’s t test, **P*<0.05). **(j)** A schematic illustration of β-catenin (left panel). β-catenin deletion mutants were overexpressed in mGFP-AQP1/HEK-293T cells and whole-cell lysates were directly subjected to Western blot using Flag antibody, β-actin was the loading control. **(k)** A schematic illustration of β-catenin (left panel). β-catenin deletion mutants were overexpressed in HEK-293T cells and whole-cell lysates were directly subjected to Western blot using Flag antibody. β-actin was the loading control. **(l)** Cell viability of AQP1/MDA-MB-231 and AQP1-ΔCT/MDA-MB-231 with different concentration of EPI treatment for 48 hrs was tested in ATP/viability assay. Values were expressed as mean ± SEM (two-tailed Student’s t test, ***P*<0.01). All Experiments were independently repeated for three times.

**Supplementary Fig. S6. AQP1 promoted β-catenin nuclear translocation. (a-b)** Immunofluorescence analysis showed the localization of β-catenin in Flag-vector/MDA-MB-231 **(a)** and Flag-AQP1/MDA-MB-231 **(b)** cells. Insets showed a high-magnification view of the indicated region. Scale bars: 50 μm. **(c)** Ratio of nuclear expression of β-catenin was reflected in a graph representation (Chi-square test, ***P*<0.01). **(d-e)** TopoIIα was knocked down in MDA-MB-231 **(d)** and Flag-AQP1/MDA-MB-231 **(e)** cells and the expression was detected by Western blot analysis, β-actin was the loading control. **(f-g)** Establishment of stable β-catenin-overexpressing MDA-MB-231 (left panel) and Flag-AQP1/MDA-MB-231 (right panel) cells through lentiviral transfection of mGFP-HA-labeled β-catenin and Western blot verification, β-actin was the loading control. **(h)** β-catenin was knocked down in MDA-MB-231 cells. The expression was detected by Western blot analysis. **(i)** β-catenin was knocked down and overexpressed in MDA-MB-231 and Flag-AQP1/MDA-MB-231 cells, respectively. The expression of β-catenin and AQP1 was detected by Western blot analysis, β-actin was the loading control. **(j)** TopoIIα deletion mutants were overexpressed in HEK-293T cells and whole-cell lysates were directly subjected to Western blot using Flag antibody. β-actin was the loading control. **(k)** Western blot analyses of TopoIIα, Flag and HA in Flag-vector/scr/HA-AQP1/MDA-MB-231 and Flag-TopoIIα-ΔCT/shTopoIIα/HA-AQP1/MDA-MB-231 cells**.** β-actin was the loading control. All Experiments were independently repeated for three times.

**Supplementary Fig. S7. Bioinformatics analysis of miR-320a-3p, AQP1 expression and anthracyclines sensitivity in breast cancer.** **(a)** The miRNA expression profile and doxorubicin sensitivity (IC_50_) data of breast cancer cell lines (n=44) were downloaded from the CCLE. Breast cancer cell lines were divided into high miR-320a-3p expression group and low miR-320a-3p expression group. The miR-320a-3p expression was found significantly negatively correlated with anthracyclines sensitivity (Mann-Whitney U test). **(b-c)** The miRNA and mRNA gene expression profiles of breast cancer tissues (n=98) were downloaded from GEO (ID: GSE19783). AQP1 expression was significantly negatively correlated with miR-320a-3p level in breast cancer **(b)**, especially in the luminal subtype **(c)** (Pearson correlation analysis).
