# Supplementary material for: Aquaporin 1 promotes sensitivity of anthracycline chemotherapy in breast cancer by inhibiting β-catenin degradation to enhance TopoIIα activity
Source: Cell Death Differ. 2020 Aug 19;28(1):382–400. doi: 10.1038/s41418-020-00607-9 (PMC7852611; doi:10.1038/s41418-020-00607-9)
Supplement: Supplementary file 10 — Supplemetary Table S2 [file 41418_2020_607_MOESM10_ESM.doc]

**Supplementary Table S2. List of plasmids, RNA interference sequences and miRNA sequences used in this study.**

| **Name** | **Sequences** |
| --- | --- |
| **PCDH-3×Flag-AQP1** |  |
| **PCDH-3×Flag-AQP1-6×Helix-CT** |  |
| **PCDH-3×Flag-AQP1-CT-delete** |  |
| **PCDH-3×Flag-mGFP-AQP1-CT** |  |
| **PCDH-3×Flag-TopoIIα-CT-delete** |  |
| **PCDH-3×Flag-TopoIIα-M** |  |
| **PCDH-3×Flag-TopoIIα-NT** |  |
| **PCDH-3×Flag-vector** |  |
| **PCDH-3×Flag-β-catenin-12×arm** |  |
| **PCDH-3×Flag-β-catenin-NT** |  |
| **PCDH-3×HA-AQP1** |  |
| **PCDH-mGFP-AQP1** |  |
| **PCDH-mGFP-HA-β-catenin** |  |
| **PCDH-mGFP-vector** |  |
| **pcDNA3.1-TopoIIα-CT-3×Flag** |  |
| **pcDNA3.1-β-catenin-CT-3×Flag** |  |
| **PLKO.1-scr** |  |
| **PLKO.1-siTopoIIα** |  |
| **PLVX-zsGreen-scr** |  |
| **PLVX-zsGreen-siβ-catenin** |  |
|  |  |
| **TopoIIα RNA interference #1** | **CAAGAAGTGTTCAGCTGTA** |
| **TopoIIα RNA interference #2** | **ACTGAATAATCAGGCTCGCTT** |
| **TopoIIα RNA interference #3** | **GCCTGATTTGTCTAAGTTTAA** |
| **TopoIIα RNA interference #4** | **GCTCCAAATCAATATGTGATT** |
| **TopoIIα RNA interference #5** | **CGCTTTCAGGGTTCTTGAGC** |
| **β-catenin RNA interference #1** | **GGATGTTCACAACCGAATTGT** |
| **β-catenin RNA interference #2** | **GCTTGGAATGAGACTGCTGAT** |
| **Negative control FAM** | **UUCUCCGAACGUGUCACGUTT** |
| **has-miR-320a-3p** | **AAAAGCUGGGUUGAGAGGGCGA** |
| **has-mirR-144-3p** | **UACAGUAUAGAUGAUGUACU** |
| **has-miR-29a-3p** | **UAGCACCAUCUGAAAUCGGUUA** |
