# Supplementary material for: Aquaporin 1 promotes sensitivity of anthracycline chemotherapy in breast cancer by inhibiting β-catenin degradation to enhance TopoIIα activity
Source: Cell Death Differ. 2020 Aug 19;28(1):382–400. doi: 10.1038/s41418-020-00607-9 (PMC7852611; doi:10.1038/s41418-020-00607-9)
Supplement: Supplementary file 11 — Supplemetary Table S3 [file 41418_2020_607_MOESM11_ESM.doc]

**Supplementary Table S3. List of antibodies used in this study.**

| **Antibodies** | **Company** | **Catalogue Number** | **Dilution** | **Species** |
| --- | --- | --- | --- | --- |
| **active β-catenin** | **EMD Millipore** | **05-665** | **WB: 1:500; IF: 1:50** | **mouse** |
| **AQP1** | **Santa Cruz Technology** | **sc-20810** | **WB: 1:1000; IF: 1:100; IHC: 1:100** | **rabbit** |
| **Axin1** | **Cell Signaling Technology** | **3323** | **WB: 1:1000** | **rabbit** |
| **CD44** | **Cell Signaling Technology** | **5670** | **WB: 1:1000** | **rabbit** |
| **C-Myc** | **Abcam** | **31072** | **WB: 1:1000** | **rabbit** |
| **CyclinD1** | **Cell Signaling Technology** | **2978** | **WB: 1:1000** | **rabbit** |
| **Flag-tag** | **Beyotime** | **AF519-1** | **WB: 1:8000; IF: 1:150** | **mouse** |
| **Flag-tag** | **Cell Signaling Technology** | **2368s** | **WB: 1:1000; IF: 1:100** | **rabbit** |
| **GSK3α/β** | **Cell Signaling Technology** | **5676** | **WB: 1:1000** | **rabbit** |
| **GSK3β** | **Cell Signaling Technology** | **12456s** | **WB: 1:1000; IF: 1:50** | **rabbit** |
| **HA-tag** | **Cell Signaling Technology** | **2368s** | **WB: 1:1000** | **rabbit** |
| **Histone** | **Ray antibody** | **RM2005** | **WB: 1:10000** | **mouse** |
| **LRP6** | **Cell Signaling Technology** | **2560** | **WB: 1:1000** | **rabbit** |
| **MMP2** | **Cell Signaling Technology** | **4022s** | **WB: 1:500** | **rabbit** |
| **phospho-β-catenin(S33/37/T41)** | **Cell Signaling Technology** | **9561** | **WB: 1:1000** | **rabbit** |
| **phospho-β-catenin(S33/37)** | **Cell Signaling Technology** | **2009** | **WB: 1:1000** | **rabbit** |
| **phospho-GSK3β(S9)** | **Cell Signaling Technology** | **9323** | **WB: 1:1000** | **rabbit** |
| **phospho-β-catenin(T41/S45)** | **Cell Signaling Technology** | **9565** | **WB: 1:1000** | **rabbit** |
| **phospho-GSK3β(Y216)** | **BD Biosciences** | **612312** | **WB: 1:1000** | **mouse** |
| **TCF4** | **EMD Millipore** | **05-511** | **WB: 1:500** | **mouse** |
| **TopoIIα** | **Santa Cruz Technology** | **sc-5348** | **WB: 1:800; IHC: 1:50** | **goat** |
| **TopoIIα** | **Santa Cruz Technology** | **sc-365918** | **IF: 1:25** | **mouse** |
| **Ub** | **Santa Cruz Technology** | **sc-271289** | **WB: 1:1000** | **mouse** |
| **β-actin** | **Santa Cruz Technology** | **sc-47778** | **WB: 1:8000** | **mouse** |
| **β-catenin** | **Santa Cruz Technology** | **7963** | **IHC: 1:50** | **mouse** |
| **β-catenin** | **Abcam** | **32572** | **WB: 1:3000; IF: 1:25** | **rabbit** |
| **β-catenin** | **BD Biosciences** | **610153** | **IF: 1:25** | **mouse** |
