# Supplementary material for: Aquaporin 1 promotes sensitivity of anthracycline chemotherapy in breast cancer by inhibiting β-catenin degradation to enhance TopoIIα activity
Source: Cell Death Differ. 2020 Aug 19;28(1):382–400. doi: 10.1038/s41418-020-00607-9 (PMC7852611; doi:10.1038/s41418-020-00607-9)
Supplement: Supplementary file 12 — Supplemetary Table S4 [file 41418_2020_607_MOESM12_ESM.doc]

**Supplementary Table S4. Primers used for RT-qPCR in this study.**

| **Gene name** | **Forward primers** | **Reverse primers** |
| --- | --- | --- |
| **AQP1** | **GTCCAGGACAACGTGAAGGT** | **GAGGAGGTGATGCCTGAGAG** |
| **CTNNB1** | **TACCTCCCAAGTCCTGTATGAG** | **TGAGCAGCATCAAACTGTGTAG** |
| **TOP2A** | **TGGCATTTTACAGCCTTCCTGA** | **AGCTTCCTTTGATGTGCTGGTG** |
| **LEF1** | **CTGCTAGAGACGCTGATCCA** | **TGGCTCTTGCAGTAGACGAA** |
| **CD44** | **CACGTGGAATACACCTGCAA** | **GACAAGTTTTGGTGGCACG** |
| **CCND1** | **TTCCTGTCCTACTACCGCCTCA** | **CCTCAGATGTCCACGTCCC** |
| **C-MYC** | **GGATTCTCTGCTCTCCTCGAC** | **CTCCAGCAGAAGGTGATCCA** |
| **TCF4** | **CCGCAACCCTCTCTAGATGT** | **TGGGATTCCTGTTTTGGGGT** |
| **MMP2** | **ACCAGCTGGCCTAGTGATGA** | **CTGGGGCAGTCCAAAGAACT** |
| **has-miR-320a-3p** | **CATGATCGTAAAAGCTGGGTTG** | **TATGGTTGTTCACGACTCCTTCAC** |
| **U6 snRNA** | **CGCTTCGGCAGCACATATAC** | **TTCACGAATTTGCGTGTCATC** |
