# Supplementary material for: Aquaporin 1 promotes sensitivity of anthracycline chemotherapy in breast cancer by inhibiting β-catenin degradation to enhance TopoIIα activity
Source: Cell Death Differ. 2020 Aug 19;28(1):382–400. doi: 10.1038/s41418-020-00607-9 (PMC7852611; doi:10.1038/s41418-020-00607-9)
Supplement: Supplementary file 13 — Supplemetary Table S5 [file 41418_2020_607_MOESM13_ESM.doc]

**Supplementary Table S5. The baseline characteristics of the 341 invasive breast cancer patients.**

| **Pathological features** | **Cases** | **Chemotherapy, n (%)** | | ***X^2^*** | ***P* value** |
| --- | --- | --- | --- | --- | --- |
|  |  | **CEF** | **non-CEF** |  |  |
| **Age** |  |  |  | **2.885** | **0.089** |
| **<50** | **341** | **52 (32.9)** | **106 (67.1)** |  |  |
| **≥50** |  | **45 (24.6)** | **138 (75.4)** |  |  |
| **Histological grade†** | **324** |  |  |  | **0.512** |
| **Grade Ⅰ** |  | **2 (18.2)** | **9 (81.8)** |  |  |
| **Grade Ⅱ** |  | **80 (31.7)** | **172 (68.3)** |  |  |
| **Grade Ⅲ** |  | **14 (23.3)** | **46 (76.7)** |  |  |
| **Grade Ⅳ** |  | **0 (0.0)** | **1 (100.0)** |  |  |
| **Tumor size, cm†** | **321** |  |  |  | **0.564** |
| **≤2** |  | **36 (32.4)** | **75 (67.6)** |  |  |
| **2-5** |  | **53 (27.0)** | **143 (73.0)** |  |  |
| **>5** |  | **3 (21.4)** | **11 (78.6)** |  |  |
| **ER status†** | **335** |  |  | **0.633** | **0.426** |
| **Negative** |  | **33 (26.4)** | **92 (73.6)** |  |  |
| **Positive** |  | **64 (30.5)** | **146 (69.5)** |  |  |
| **PR status†** | **335** |  |  | **2.389** | **0.122** |
| **Negative** |  | **25 (23.4)** | **82 (76.6)** |  |  |
| **Positive** |  | **72 (31.6)** | **156 (68.4)** |  |  |
| **HER2 status†** | **339** |  |  | **0.468** | **0.494** |
| **Negative** |  | **79 (29.5)** | **189 (70.5)** |  |  |
| **Positive** |  | **18 (25.4)** | **53 (74.6)** |  |  |

**† Some missing data**
