# Supplementary material for: Aquaporin 1 promotes sensitivity of anthracycline chemotherapy in breast cancer by inhibiting β-catenin degradation to enhance TopoIIα activity
Source: Cell Death Differ. 2020 Aug 19;28(1):382–400. doi: 10.1038/s41418-020-00607-9 (PMC7852611; doi:10.1038/s41418-020-00607-9)
Supplement: Supplementary file 14 — Supplemetary Table S6 [file 41418_2020_607_MOESM14_ESM.doc]

**Supplementary Table S6. The baseline characteristics of the breast cancer patients with AQP1 low expression.**

| **Pathological features** | **Cases** | **Chemotherapy, n (%)** | | | ***X^2^*** | ***P* value** |
| --- | --- | --- | --- | --- | --- | --- |
|  |  | **CEF** | **CMF** | **Other** |  |  |
| **Age** | **198** |  |  |  | **4.092** | **0.129** |
| **<50** |  | **35 (36.5)** | **12 (12.5)** | **49 (51.0)** |  |  |
| **≥50** |  | **25 (24.5)** | **20 (19.6)** | **57 (55.9)** |  |  |
| **Histological grade†** | **191** |  |  |  |  | **0.220** |
| **Grade Ⅰ** |  | **1 (14.3)** | **3 (42.9)** | **3 (42.9)** |  |  |
| **Grade Ⅱ** |  | **52 (33.5)** | **22 (14.2)** | **81 (52.3)** |  |  |
| **Grade Ⅲ** |  | **7 (24.1)** | **7 (24.1)** | **15 (51.7)** |  |  |
| **Tumor size, cm†** | **189** |  |  |  |  | **0.672** |
| **≤2** |  | **19 (30.2)** | **13 (20.6)** | **31 (49.2)** |  |  |
| **2-5** |  | **36 (31.3)** | **17 (14.8)** | **62 (53.9)** |  |  |
| **>5** |  | **2 (18.2)** | **1 (9.1)** | **8 (72.7)** |  |  |
| **ER status†** | **193** |  |  |  | **2.160** | **0.374** |
| **Negative** |  | **18 (27.3)** | **14 (21.2)** | **34 (51.5)** |  |  |
| **Positive** |  | **42 (33.1)** | **17 (13.4)** | **68 (53.5)** |  |  |
| **PR status†** | **193** |  |  |  | **5.379** | **0.068** |
| **Negative** |  | **13 (22.4)** | **14 (24.1)** | **31 (53.4)** |  |  |
| **Positive** |  | **47 (34.8)** | **17 (12.6)** | **71 (52.6)** |  |  |
| **HER2 status†** | **197** |  |  |  | **1.160** | **0.560** |
| **Negative** |  | **47 (29.4)** | **28 (17.5)** | **85 (53.1)** |  |  |
| **Positive** |  | **13 (35.1)** | **4 (10.8)** | **20 (54.1)** |  |  |

**† Some missing data**
