# Supplementary material for: Aquaporin 1 promotes sensitivity of anthracycline chemotherapy in breast cancer by inhibiting β-catenin degradation to enhance TopoIIα activity
Source: Cell Death Differ. 2020 Aug 19;28(1):382–400. doi: 10.1038/s41418-020-00607-9 (PMC7852611; doi:10.1038/s41418-020-00607-9)
Supplement: Supplementary file 15 — Supplemetary Table S7 [file 41418_2020_607_MOESM15_ESM.doc]

**Supplementary Table S7. The baseline characteristics of the breast cancer patients with AQP1 high expression.**

| **Pathological features** | **Cases** | **Chemotherapy, n (%)** | | | ***X^2^*** | ***P* value** |
| --- | --- | --- | --- | --- | --- | --- |
|  |  | **CEF** | **CMF** | **other** |  |  |
| **Age** | **143** |  |  |  | **0.615** | **0.735** |
| **<50** |  | **17 (27.4)** | **13 (21.0)** | **32 (51.6)** |  |  |
| **≥50** |  | **20 (24.7)** | **14 (17.3)** | **47 (58.0)** |  |  |
| **Histological grade†** | **133** |  |  |  |  | **0.939** |
| **Grade Ⅰ** |  | **1 (25.0)** | **1 (25.0)** | **2 (50.0)** |  |  |
| **Grade Ⅱ** |  | **28 (28.9)** | **20 (20.6)** | **49 (50.5)** |  |  |
| **Grade Ⅲ** |  | **7 (22.6)** | **5 (16.1)** | **19 (61.3)** |  |  |
| **Grade Ⅳ** |  | **0 (0.0)** | **0 (0.0)** | **1 (100%)** |  |  |
| **Tumor size, cm†** | **132** |  |  |  |  | **0.390** |
| **≤2** |  | **17 (35.4)** | **8 (16.7)** | **23 (47.9)** |  |  |
| **2-5** |  | **17 (21.0)** | **16 (19.8)** | **48 (59.3)** |  |  |
| **>5** |  | **1 (33.3)** | **0 (19.8)** | **2 (66.7)** |  |  |
| **ER status†** | **142** |  |  |  | **0.041** | **0.980** |
| **Negative** |  | **15 (25.4)** | **11 (18.6)** | **33 (55.9)** |  |  |
| **Positive** |  | **22 (26.5)** | **16 (19.3)** | **45 (54.2)** |  |  |
| **PR status†** | **142** |  |  |  | **1.490** | **0.475** |
| **Negative** |  | **12 (24.5)** | **7 (14.3)** | **30 (61.2)** |  |  |
| **Positive** |  | **25 (26.9)** | **20 (21.5)** | **48 (51.6)** |  |  |
| **HER2 status†** | **142** |  |  |  | **4.642** | **0.098** |
| **Negative** |  | **32 (29.6)** | **22 (20.4)** | **54 (50.0)** |  |  |
| **Positive** |  | **5 (14.7)** | **5 (14.7)** | **24 (70.6)** |  |  |

**† Some missing data**
