# Supplementary material for: Aquaporin 1 promotes sensitivity of anthracycline chemotherapy in breast cancer by inhibiting β-catenin degradation to enhance TopoIIα activity
Source: Cell Death Differ. 2020 Aug 19;28(1):382–400. doi: 10.1038/s41418-020-00607-9 (PMC7852611; doi:10.1038/s41418-020-00607-9)
Supplement: Supplementary file 16 — Supplemetary Table S8 [file 41418_2020_607_MOESM16_ESM.doc]

**Supplementary Table S8. Correlations among the expression of AQP1, β-catenin and TopoIIα.**

| **Variable**  **(rate of high expression)** | **AQP1**  **(38/70, 54.3%)** | **β-catenin**  **(54/70, 77.1%)** | **TopoIIα**  **(26/70, 37.1%)** |
| --- | --- | --- | --- |
| **AQP1** |  | ***r*_s_ =0.457**  ***P*= 0.000**  **(36/70, 51.4%)** | ***r*_s_ =0.231**  ***P*= 0.055**  **(18/70, 25.7%)** |
| **β-catenin** |  |  | ***r*_s_ =0.207**  ***P*=0.085**  **(23/70, 32.9%)** |

***P* value was calculated by Spearman’s Rank-Correlation test.**
